# Large-scale analysis of cell-cell communication reveals angiogenin-dependent tumor progression in clear cell renal cell carcinoma

**DOI:** 10.1016/j.isci.2023.108367

**Published:** 2023-10-31

**Authors:** Lucile Massenet-Regad, Justine Poirot, Margaret Jackson, Caroline Hoffmann, Elise Amblard, Fanny Onodi, Fatiha Bouhidel, Malika Djouadou, Idir Ouzaid, Evanguelos Xylinas, Jasna Medvedovic, Vassili Soumelis

**Affiliations:** 1 Université Paris Cité, INSERM, U976 HIPI, F-75010 Paris, France; 2 Université Paris-Saclay, F-91190 Saint Aubin, France; 3 INSERM U932, Department of Surgical Oncology, PSL University, Institut Curie, 75005 Paris, France; 4 Owkin France, 75010 Paris, France; 5 CNRS, UMR 5525, VetAgro Sup, Grenoble INP, TIMC, Grenoble Alpes University, 38000 Grenoble, France; 6 Department of Pathology, Saint-Louis Hospital, AP-HP.Nord, Université Paris Cité, 75010 Paris, France; 7 Department of Urology, Saint-Louis Hospital, AP-HP.Nord, Université Paris Cité, 75010 Paris, France; 8 Department of Urology, Bichat-Claude Bernard Hospital, AP-HP.Nord, Université Paris Cité, 75018 Paris, France; 9 Department of Immunology-Histocompatibility, Saint-Louis Hospital, AP-HP.Nord, Université Paris Cité, 75010 Paris, France

**Keywords:** Microenvironment, Cancer, Transcriptomics

## Abstract

Cellular crosstalk in the tumor microenvironment (TME) is still largely uncharacterized, while it plays an essential role in shaping immunosuppression or anti-tumor response. Large-scale analyses are needed to better decipher cell-cell communication in cancer. In this work, we used original and publicly available single-cell RNA sequencing (scRNAseq) data to characterize in-depth the communication networks in human clear cell renal cell carcinoma (ccRCC). We identified 50 putative communication channels specifically used by cancer cells to interact with other cells, including two novel angiogenin-mediated interactions. Expression of angiogenin and its receptors was validated at the protein level in primary ccRCC. Mechanistically, angiogenin enhanced ccRCC cell line proliferation and down-regulated secretion of IL-6, IL-8, and MCP-1 proinflammatory molecules. This study provides novel biological insights into molecular mechanisms of ccRCC, and suggests angiogenin and its receptors as potential therapeutic targets in clear cell renal cancer.

## Highlights

• Single-cell RNA sequencing delineates ccRCC cancer cell-specific vocabulary
• CcRCC cancer cells use specific communication channels to modulate the TME
• Angiogenin (ANG) and its receptors EGFR and PLXNB2 are upregulated by cancer cells
• ANG-dependent channels enhance cell growth and reduce IL-8, IL-6 and MCP-1 secretion

## Graphical abstract

**Figure:**
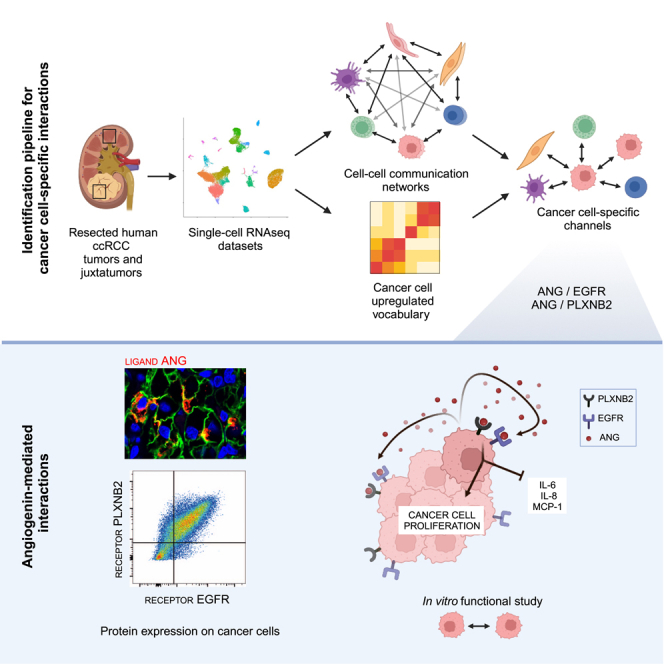

## Introduction

In the tumor microenvironment (TME), tumoral, immune, endothelial, and stromal cells express a set of communication molecules that may modulate their surroundings. Depending on the cell states and biological context, cellular crosstalk can either favor tumor growth, immunosuppression or participates in the anti-tumor response.^1^ Communication molecules can be blocked by monoclonal antibodies or administered in recombinant forms, such as PD-1 checkpoint blockade and IL-2 treatments,^2^ offering significant therapeutic potential. Therefore, comprehensive analyses of the intercellular communication networks are essential and may lead to the development of new therapeutic strategies.

In clear cell renal cell carcinoma (ccRCC), current treatments options do improve overall survival of some metastatic patients.^3^ However, they are ineffective in most patients, and there is a need for the identification of new therapeutic targets or relevant combinations of treatments. In ccRCC, current effective systemic therapies include tyrosine-kinase inhibitors (TKI) and more recently PD-1 blockade.^4^ These therapeutic agents directly target cell-cell communication by blocking specific ligand-receptor interactions or limiting their downstream signaling, suggesting that cell-cell communication is important for treatment in ccRCC. Also, ccRCC is among the most immune-infiltrated cancer types.^5^ This suggests prominent cell-cell communication, as immune cell functions highly rely on communication signals, which are also needed to coordinate an anti-tumor response.

To date, cell-cell communication in ccRCC has been investigated using a diversity of experimental and *in silico* approaches, revealing key communication molecules and signaling pathways activated in the TME.^6^^,^^7^^,^^8^ However, most of these experimental studies devoted to deciphering cellular communication mechanisms are limited to a few molecules or focused on a single cellular crosstalk.^9^^,^^10^ Similarly, bulk RNAseq lacks cell resolution and offers limited comprehension of communication mechanisms. Recent methods have been developed to infer cell-cell communication on a large scale based on single-cell transcriptomic expression of ligand and receptor genes.^11^^,^^12^^,^^13^ These methods notably highlighted major tumor-stoma and tumor-immune interactions,^14^^,^^15^^,^^16^ interactions modulated when exposed to PD-1 blockade^17^^,^^18^ or upon *VHL* mutation,^19^ and molecules potentially driving tumor progression.^16^^,^^20^ However, the inferred ligand-receptor interactions often lack experimental validation at the protein level and the assessment of their biological function. Large-scale and comprehensive analyses considering all cell types in the TME to identify new therapeutic targets are still needed.

In this study, we comprehensively characterized cell-cell communication networks in ccRCC tumors using publicly available and original single-cell RNA sequencing (scRNAseq) datasets. We identified and validated interactions specifically used by cancer cells to interact with the other cells of the TME and suggested new therapeutic targets in ccRCC.

## Results

### Exhaustive mapping of ligand-receptor interactions in the clear cell renal cell carcinoma tumor microenvironment

**Figure 1:**
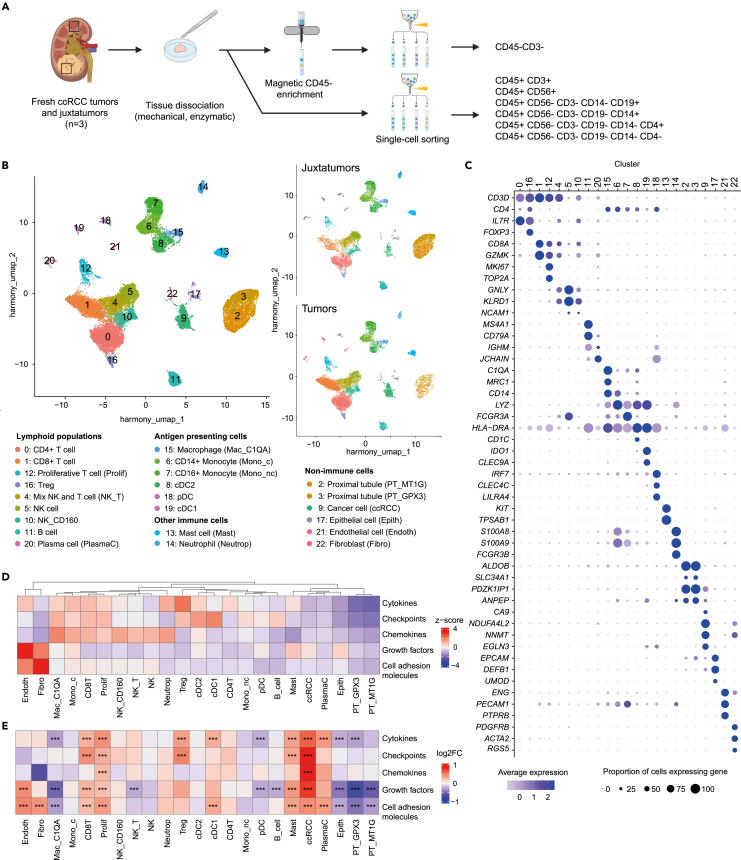
Landscape of communication molecule expression in clear cell renal cell carcinoma (A) Experimental strategy for scRNAseq data generation from 3 freshly resected ccRCC tumors and juxtatumors. After mechanical and enzymatic dissociation, a fraction of the cell suspension was enriched in CD45^−^cells. All fractions were then stained to sort specific cell populations before sequencing. Cell sorting gating strategy is provided in Figure S1. Illustrations created with BioRender.com. (B) Uniform Manifold Approximation and Projection (UMAP) visualization of the original scRNAseq (n = 3 patients, tumors and juxtatumors), pooled together (left) or displayed separately according to tissue of origin (right), colored according to cluster identity. From the 27,963 pre-processed cells, 23 clusters were identified and manually annotated. NK: natural killer; Treg: regulatory T cell; cDC: conventional dendritic cell; pDC: plasmacytoid dendritic cell. (C) Expression levels of selected genes used for the cell type assignment of the clusters. Color corresponds to the intensity of average expression, and dot size to the percentage of cells expressing the gene in the cluster. (D) Global expression score computed for each family of molecules (cytokines, immune checkpoints, chemokines, growth factors and cell adhesion molecules) considering cells from tumor samples only, grouped by cell types (average expression profile of the cell type). For each family of molecules, global expression scores were centered-reduced as z-scores. Cell types were ordered according to Ward hierarchical clustering using Euclidean distance. (E) Differences of global expression score between cells from tumors and juxtatumors, considering separately each cell type (average expression profile of the cell type in each tissue) and each family of molecules. Significant differences according to Wilcoxon tests with FDR correction and with log2 fold change (log2FC) greater than 0.25 are highlighted with stars. ∗∗∗: p value <0.001.See also Figure S1, Tables S1, and S2.

In order to investigate cell-cell communication in the ccRCC TME, we generated a scRNAseq dataset from 3 fresh human primary ccRCC, analyzing tumor tissue and adjacent healthy tissue, herein referred to as “juxtatumor” (Figure 1A). Before sequencing, cell types were sorted by flow cytometry to capture and profile rare cell types in the TME, such as dendritic cells (Figures S1A and S1B), and to enrich for cells challenging to recover from human tissue such as CD45^−^cells. After quality controls and integration of the samples to remove batch effect, the dataset included 27,963 cells split into 23 clusters shared by all the patients and found in both tissues (Figure 1B; Figures S1C and S1D). Cell identities were assigned manually using known markers from the literature (Figure 1C; Table S1). The dataset included all major cell types expected in the ccRCC TME from the malignant, stromal, vascular, and immune compartments (Figure 1B; Table S2). In addition to expected immune, vascular and stromal cell types in juxtatumors, we identified a small number of CD45^−^cells displaying a malignant cell-like phenotype at the transcriptomic level, although no CD45-CA9+ cells were measured by flow cytometry (Figure S1A).

To consider all possible ligand-receptor interactions that could play a key role in ccRCC TME, we manually extended the previously developed ICELLNET ligand-receptor interaction database.^11^ Compared to its original version, we added all interactions involving communication molecules differentially expressed in at least one cluster, by comparing the transcriptome of cells from tumoral samples with those from juxtatumoral samples (see STAR methods). Newly added communication molecules were identified using the most comprehensive list of ligand-receptor interaction database available, NATMI,^21^ and cognate interactions were manually curated from the literature. In contrast to NATMI ligand-receptor database, we included ligand and receptor heterodimers, and retained only interactions demonstrated experimentally in a human system. Putative interactions based on protein-protein predictions were excluded. In addition to the 752 interactions originally included in ICELLNET,^11^^,^^22^ we integrated 412 interactions, making a total of 1,164 ligand-receptor interactions (Table S3).

### Expression of communication molecules in the clear cell renal cell carcinoma tumor microenvironment is cell type-specific

In order to get an initial overview of how cells interact, we asked how communication molecules expression was distributed in ccRCC TME. To this end, we computed a global expression score gathering the expression of genes classified in broad families of communication molecules within the ICELLNET database, including 253 cytokines, 76 immune checkpoints, 73 chemokines, 94 growth factors, and 122 cell adhesion molecules. Global expression scores were computed by adding the normalized counts of all ligand and receptor genes belonging to the same family of molecules, grouping cells by clusters and their tissue of origin (Figure 1D) (see STAR methods). Based on the analysis of tumor samples only, endothelial cells (Endoth) and fibroblasts (Fibro) were found to be the main sources and targets of growth factors and cell adhesion molecules in the TME. Ligands and receptors belonging to cytokine, immune checkpoint and chemokine families were more broadly expressed by almost all immune cell populations at similar levels, and at higher levels than fibroblasts, cancer cells (RCC cluster), proximal tubules (PT_GPX3 and PT_MT1G) and epithelial cells (Epith).

We then assessed differences in expression scores between cells from tumors and juxtatumors by Wilcoxon tests and filtered for significant differences (p < 0.001), with a minimum absolute log2-fold change of 0.25 (Figure 1E). Similarly, cancer cells were compared to proximal tubules (PT) from juxtatumor samples, as PT were previously identified as the cancer cell type of origin by previous studies.^14^^,^^23^ Overall, 9 cell types significantly over-expressed at least one family of molecules in tumors, whereas 7 cell types significantly under-expressed at least one family of molecules in tumors. Among cell types up-regulating communication molecules in tumors, cancer cells, CD8^+^ T lymphocytes (CD8T), regulatory T cell (Treg), proliferative T cells and mast cells (Mast) significantly over-expressed several families of molecules. Cancer cells displayed the highest increase of global expression of growth factor, chemokine, immune checkpoint, and cytokine families compared to other cell types. Conversely, macrophage (Mac_C1QA), plasmacytoid dendritic cells (pDC), epithelial cells and PT significantly down-regulated cytokine, cell adhesion and growth factor families in tumors. No major changes were observed for the remaining clusters, corresponding to monocytes (Mono_c, Mono_nc), NK clusters, cDC2, and neutrophils (Neutrop). This analysis suggests a substantial modulation of the cellular crosstalk induced by cancer cells in the TME.

### Identification of clear cell renal cell carcinoma cancer cell-specific vocabulary in the tumor microenvironment

**Figure 2:**
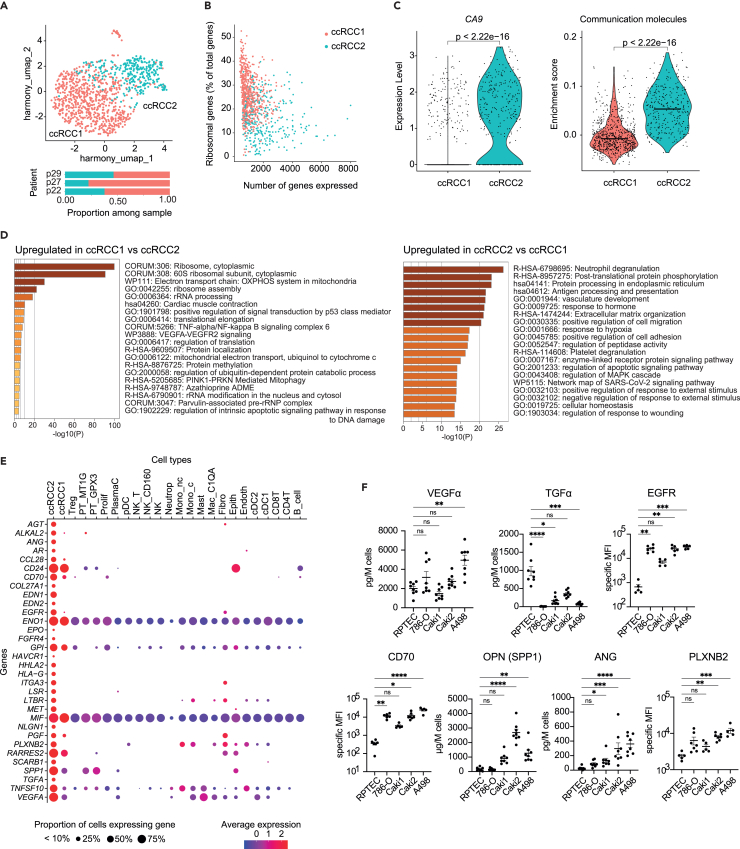
Characterization of ccRCC cancer cell-specific vocabulary within the TME (A) UMAP of the cancer cells from tumor samples. Two sub-clusters were identified at a resolution of 0.1: ccRCC1 (pink), and ccRCC2 (blue). Proportions of each sub-cluster among the 3 patients are represented later in discussion the UMAP. (B) Proportion of expressed genes belonging to the ribosomal gene family (in percentage), in the function of the total number of expressed genes, for each cancer cell subcluster. (C) Expression of *CA9* (ccRCC cancer cell marker) for each cancer cell subcluster (left), and enrichment score of communication genes in each cancer cell subcluster (right). The scores were compared by Wilcoxon tests. (D) Functional enrichment analysis of ccRCC1 compared to ccRCC2 cells (left), or conversely (right). Genes differentially expressed between ccRCC1 and ccRCC2 cells and filtered for minimum log2FC of 0.5 and p < 0.05 were considered. See also Table S4. (E) Expression of genes significantly upregulated by ccRCC2 compared two-by-two to all other non-tumoral cell clusters of the TME. Color corresponds to the intensity of average expression, and dot size to the percentage of cells expressing the gene in the cluster. (F) Expression of VEGFα, EGFR, TGFα, ANG, CD70, OPN, PLXNB2 by two ccRCC cell lines (786-O, Caki2), and a proximal tubule primary cell line (RPTEC), at protein levels. Expression of surface markers is quantified by flow cytometry using specific mean fluorescence intensity (MFI), corresponding to the MFI obtained with specific antibody subtracted by those with the corresponding isotype (n = 5 up to 7 biologically independent replicates). Secreted molecules were measured from cell lines supernatants (n = 8 biologically independent replicates). Data are represented as mean values ±SEM. Conditions were compared using Kruskal–Wallis statistical tests combined with a Dunn’s post-hoc. ns, not significant; ∗p < 0.05; ∗∗p < 0.01; ∗∗∗p < 0.001; ∗∗∗∗p < 0.0001. See also Figure S2.

We then focused on the malignant cells coming from tumoral samples, in order to identify communication molecules specifically over-expressed by cancer cells in the TME. Malignant cells (cluster 9) expressed ccRCC markers mentioned in the literature such as *CA9, NNMT, and NDUFA4L2*.^14^^,^^23^ Sub-clustering of malignant cells led to the identification of two sub-clusters (ccRCC1, ccRCC2) shared by all patients (Figure 2A). ccRCC1 cells expressed considerably fewer genes and higher proportion of ribosomal genes than ccRCC2, suggesting damaged or inactive cells (Figure 2B; Figure S2A). Strikingly, ccRCC2 expressed significantly higher levels of *CA9* and communication molecules (Figure 2C). Functional enrichment analysis based on differentially expressed genes between ccRCC1 and ccRCC2 cells revealed that ccRCC2 cells were more involved in cell-cell communication signaling and pro-tumoral processes, such as “post-translational protein phosphorylation,” “response to hormone,” “vasculature development,” “positive regulation of cell migration” modules, whereas ccRCC1 cells were highly enriched in ribosomal activity-related modules (Figure 2D; Table S4). Together, these results led us to focus on ccRCC2 cells to investigate cancer cell-mediated communication.

We then identified communication molecules specifically expressed by cancer cells in the TME, by performing a differential expression analysis of communication molecules between ccRCC2 and each non-malignant cell type of the TME individually. Thirty-two molecules were specifically expressed by ccRCC2 cells (Figure 2D). Among them, using four ccRCC cell lines (786-O, Caki1, Caki2, A498), we experimentally verified the expression at protein level of molecules known to be preferentially expressed by ccRCC cells, VEGFα, TGFα, and EGFR (Figure 2E). We were also able to confirm the expression of 4 additional molecules CD70, osteopontin (OPN, encoded by *SPP1* gene), ANG and PLXNB2. Five out of these molecules were significantly more expressed by Caki2 and A498 cells as compared to primary proximal tubular cells RPTEC. EGFR and CD70 were also significantly more expressed by Caki1 and 786-O cells compared to RPTEC, while similar levels were found for OPN, VEGFα, and PLXNB2 (Figure 2F).

Hence, we were able to identify thirty-two ligand or receptor genes specifically expressed by the cancer cells, some genes being involved in cell proliferation, angiogenesis and immunosuppression. We suggest that these genes collectively form ccRCC cells specific vocabulary.

### Clear cell renal cell carcinoma cancer cells use specific communication channels

**Figure 3:**
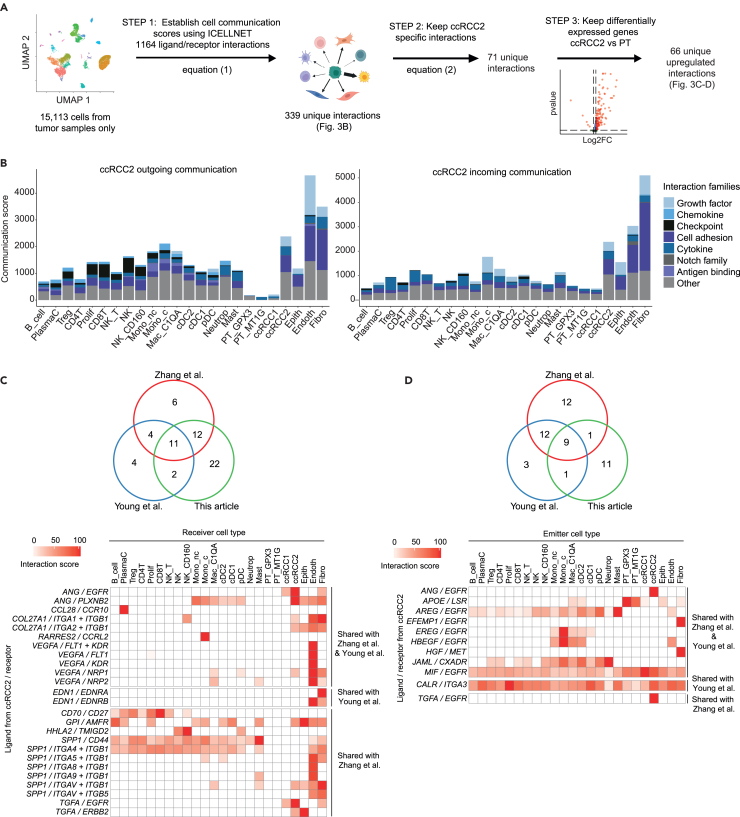
Identification of communication channels specifically used by cancer cells to interact with ccRCC TME (A) Description of the cell-cell communication analysis to identify ccRCC2 specific interactions in the TME and upregulated as compared to proximal tubules (PT). Equations 1 and 2 are detailed in STAR methods. (B) Barplot representing global communication scores between ccRCC2 and the other cell types included in the scRNAseq dataset. (left) represents the outgoing communication scores, meaning ligand expressed by ccRCC2 and receptors expressed by the other cell types. Conversely, (right) represents the incoming communication scores. The contribution of each family of molecules to the communication scores is represented by the color code. See also Figure S2. (C and D) Intersection of cancer cell-specific ligand/receptor interactions in 3 datasets. (C) represents outgoing communication (ccRCC2 as emitter cell), and (D) incoming communication (ccRCC2 as receiver cell). Based on original dataset, shared interactions between the original and public datasets are represented at the bottom as an heatmap. The heatmap displays the interaction score (from 0 to 100) between ccRCC2 and the other cell type (horizontal axis). See also Figures S3 and S4 and Table S6.

We next sought to dissect the connectivity of cancer cells with the other cell types of the TME. Cell-cell communication networks between cancer cells and other cell types were investigated using ICELLNET,^11^ which computes a communication score between cell types based on the product of their average expression profiles (see STAR methods) (Figure 3A, Step 1).

Considering ccRCC2 as emitter (outgoing communication), the highest communication scores were seen with endothelial cells, fibroblasts, and themselves, with the high representation of growth factors and cell adhesion interactions (Figure 3B). Conversely, ccRCC2 mostly received communication (incoming communication) signals from fibroblasts, followed by endothelial cells and themselves. The same analysis using ccRCC1 cells as emitter or receiver cell confirmed that ccRCC1 barely communicate with all cell types of the TME compared to ccRCC2 (Figure S2B).

Among the ligand-receptor interactions used by cancer cells, we focused on those specifically used by cancer cells to interact with other cell types in the TME. It included interactions: 1) for which the communication score between the ccRCC2 and another cell type was 1.5 times higher than all the other scores between cell pairs (Figure 3A, Step 2), and 2) upregulated by the ccRCC2 cancer cells as compared to PT from juxtatumors (Figure 3A, Step 3; Table S5). This led us to select 66 ligand/receptor putative interactions (Table S6). Almost all of them were specific to ccRCC2 cells, as compared to the same analysis using ccRCC1 cells (Figure S2C).

We applied the same strategy to explore two publicly available ccRCC scRNAseq (Figures S2A–S2C). Notably, 11 outgoing and 9 incoming interactions were identified in the 3 datasets as specifically used by the cancer cells to interact within the TME (Figures 3C and 3D; Figures S3B and S3C; Table S6). Thirty additional interactions were shared by at least two datasets. Most of the identified interactions are involved in angiogenesis (such as *CCL28/CCR10*, *or VEGFA/FLT1 + KDR*)^24^ or known to play a role in malignant and stromal cell proliferation (such as *EDN1/EDNRB* with endothelial cells, *EDN1/EDNRA* with fibroblasts, and all interactions targeting *EGFR* on cancer cells).^25^^,^^26^ Other interactions are described to be involved in the regulation of leukocyte migration (*RARRES2/CCRL2, COL1A1/DDR1*),^27^^,^^28^ immunosuppression (*CD24/SIGLEC10* with antigen-presenting cells)^29^ or immune regulation (*HHLA2/TMIGD2, CD70/CD27*).^30^^,^^31^ Most of these interactions were barely used by ccRCC1 cells in comparison to ccRCC2 cells (Figures S2D and S2E). Four interactions were found to be specifically used by ccRCC1 only, but they were not identified in the two other datasets (Figure S2C).

We then validated our results with a different computational method, CellChat,^32^ which uses law of mass action and consider ligands, receptors, agonist and antagonist molecules to infer cell-cell communication probabilities. CellChat also offers metrics to identify key receiver or emitter cell types. Fibroblasts, endothelial cells and ccRCC2 were the top three main emitter cell types, confirming that ccRCC2 has a substantial role in modulating cell crosstalk in ccRCC TME (Figure S4A). From the 50 previously identified interactions, 20 were also predicted with CellChat, with highest probabilities outgoing from ccRCC2 (Figures S4B and S4C). The 30 remaining interactions could not be confirmed with CellChat, as these ligand-receptor pairs were not included in the CellChat database. This validates the performance of ICELLNET to infer cell-cell communication networks and shows the importance of the comprehensive mapping of ligand-receptor interactions using data-driven and biological knowledge.

Although single-cell technologies allow for the exploration of tumors at high resolution, scRNAseq datasets usually include a low number of samples which may not be representative of the disease heterogeneity. We thus sought to explore the expression of the 50 previously mentioned interactions on a larger cohort, the TCGA-KIRC bulk RNAseq data. For each interaction, we computed the product of ligand and receptor expression in each sample (Figure S4D, see STAR methods). Twenty-eight interactions were significantly over-expressed (p < 0.001) in tumors (n = 538 samples) compared to normal tissues (n = 72 samples), with a minimum log2-fold change of 1.

For downstream experimental validations and mechanistic studies, we thus selected interactions identified both by ICELLNET on scRNAseq datasets and upregulated in TCGA-KIRC (Table S6). Systematic screening of the 28 interactions in the literature led to focus on *ANG/EGFR* and *ANG/PLXNB2* interactions due to their potential clinical impact. In the TME, *ANG* is expressed by cancer cells, *EGFR* is expressed mostly by cancer cells and to a lesser extent by fibroblasts, and *PLXNB2* is expressed mainly by dendritic cells, macrophages, and cancer cells (Figures 3C and 3D). For both interactions, the highest communication scores were obtained between ccRCC2 and themselves, suggesting two putative tumor cell autocrine loops.

### Angiogenin and its receptors estimated glomerular filtration rate and PLXNB2 are upregulated by clear cell renal cell carcinoma cells in renal tumor microenvironment

**Figure 4:**
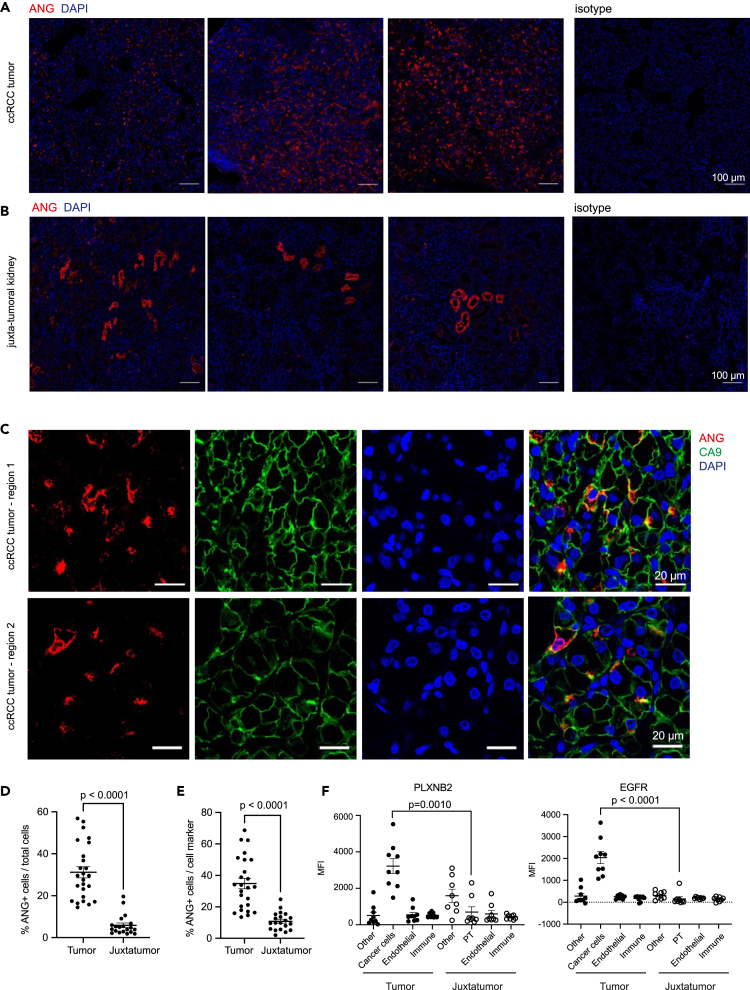
Angiogenin, EGFR and Plexin-B2 protein expression by ccRCC cancer cells (A and B) Angiogenin (red) tissue distribution in three representative ccRCC tumors (A) or juxtatumors (B) by immunofluorescence. Scale bar corresponds to 100 μm. An isotype anti-IgG is used as a control of anti-ANG antibody specificity. See also Figure S5. (C) ANG (red) and CA9 (green) expression on ccRCC human tumors. Scale bar corresponds to 20 μm. (D) Quantification of ANG positive cells normalized by total number of cells in ccRCC tumors (n = 25 images from 12 biologically independent samples) or juxtatumors (n = 19 images from 10 biologically independent samples). Data are represented as mean values ±SEM. Conditions were compared using Mann-Whitney statistical test. (E) Quantification of ANG positive cells normalized by CA9+ positive cells in tumors or PDZK1IP1+ cells in juxtatumors. Data are represented as mean values ±SEM. Conditions were compared using Mann-Whitney statistical test. (F) Surface expression of PLXNB2 (left) or EGFR (right) by immune cells (CD45^+^), endothelial cells (CD45^−^CD31^+^), cancer cells or PT (CD45^−^CD31^−^CD13^+^), and other cells (CD45^−^CD31^−^CD13^−^) in human ccRCC tumors and juxtatumors (n = 9 and n = 8 biologically independent samples). Expression is quantified as specific mean fluorescence intensity (MFI). Data are represented as mean values ±SEM. Conditions were compared using Kruskal–Wallis statistical tests combined with a Dunn’s post-hoc. Gating strategy is provided in Figure S5.

We validated the expression of angiogenin at the protein level on ccRCC tumors and juxtatumors by immunofluorescence (n = 13 tumors and n = 10 juxtatumors). Angiogenin was found to be expressed in both tissues (Figures 4A and 4B), but with different distributions. In juxtatumors, ANG expression was restricted to certain groups of PT (Figure S5B), whereas ANG was more widely expressed in ccRCC tumor areas. In tumoral samples, angiogenin signal mostly colocalized with CA9 staining, used here as a marker of cancer cells (Figure 4C; Figure S5A). Quantification of the images showed that the proportion of ANG-positive cells increased in ccRCC tumors as compared to juxtatumors (Figure 4D). We also compared the proportion of ANG-positive cells among PT in juxtatumors (CD13 positive cells) and cancer cells in tumors (CA9 positive cells). ANG was more frequently expressed by cancer cells in tumors than PT in juxtatumors (Figure 4E). The expression of EGFR and PLXNB2 receptors were also validated at protein level by flow cytometry on freshly resected ccRCC tumors and juxtatumors (n = 9 tumors, n = 8 juxtatumors) in immune, malignant, endothelial, and stromal cell populations (Figures S5C and S5D). In tumors, both receptors were mostly expressed by cancer cells and their expression was significantly increased relative to expression by PT in juxtatumors samples (Figure 4F). EGFR and PLXNB2 co-expression was widely observed in malignant cells, in average by almost 78 ± 3% (mean values ±SEM) of them (Figures S5E and S5F).

### Angiogenin silencing impairs cancer cell proliferation and increases pro-inflammatory cytokine secretion

**Figure 5:**
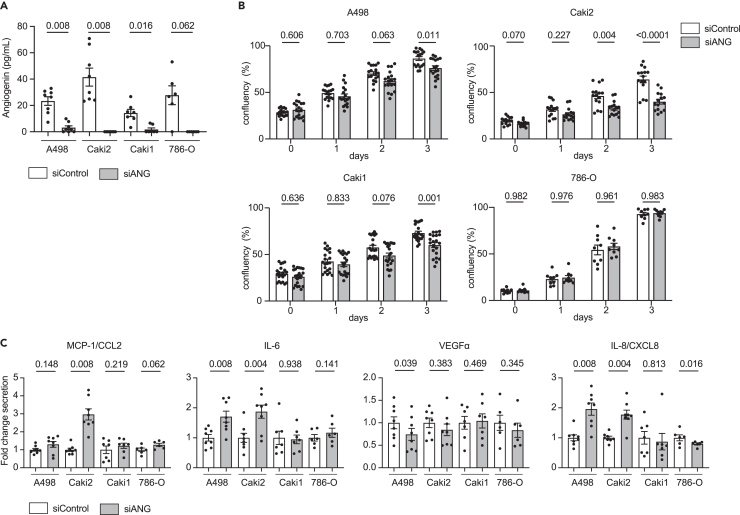
Angiogenin silencing *in vitro* inhibits cancer cell proliferation and increases pro-inflammatory molecules secretion (A) Secretion levels of ANG of Caki1, Caki2, A498 and 786-O cell lines upon ANG silencing (siANG) or control (siControl) condition (biologically independent replicates, n = 8 for Caki1, Caki2 and A498, n = 6 for 786-O). Conditions were compared using Wilcoxon matched-pairs signed rank tests. Data are presented as mean values ±SEM. (B) Analysis of Caki1, Caki2, A498 and 786-O cell proliferation after transfection with siRNA against ANG (siANG) or control condition (siControl). The proliferation is measured by Incucyte to quantify cell confluency over time. Each independent experiments were run in triplicates (n = 6 independent experiments for A498 and Caki2, n = 7 for Caki1, and n = 3 for 786-O). Data are presented as mean values ±SEM. Conditions were compared using repeated measures two-way ANOVA with Geisser Greenhouse correction combined with a Šidák post hoc. See also Figure S6. (C) Secretion levels IL-6, IL-8, MCP-1 (CCL2) and VEGF-α for Caki2 and 786-O cell lines upon siControl or siANG perturbation (biologically independent replicates, n = 8 for Caki1, Caki2 and A498, n = 6 for 786-O). Data are presented as mean values ±SEM. Conditions were compared using Wilcoxon matched-pairs signed rank tests.

Previous studies have demonstrated that angiogenin is involved in cell proliferation, notably of endothelial cells.^33^ We thus downregulated the expression of angiogenin in four ccRCC cell lines, 786-O, Caki1, Caki2, and A498 using siRNA to investigate the biological functions of angiogenin in cancer cells. siRNA treatment significantly decreased angiogenin expression in all cell lines (Figure 5A) and did not impact EGFR and PLXNB2 expression (data not shown). Angiogenin knock-down significantly impaired cancer cell proliferation on three out of the four cell lines tested (Figure 5B; Figure S6).

We then investigated how angiogenin silencing would impact the secretion of soluble factors expressed by cancer cells. We first assessed the expression levels of very well-known pro-inflammatory molecules, such as TNFα, IL1-b, IL-6, and MCP-1, and molecules involved in angiogenesis, IL-8 and VEGFα, described to be regulated by angiogenin in the literature.^34^^,^^35^

Angiogenin knock-down was associated with the increased secretion of MCP-1 and IL-6 in Caki2, A498 and 786-O cells, and increased expression of IL-8 in Caki2 and A498 cells (Figure 5C). VEGFα levels were similar in both conditions for most cell lines, except for A498 where lower levels of VEGFα were measured upon siRNA treatment. TNFα and IL-1β were not detected in the cell lines supernatants. Our results thus suggest that angiogenin enhances cancer cell proliferation and down-regulates the secretion of pro-inflammatory molecules.

## Discussion

The scRNAseq data generated and subsequent analyses revealed 32 specific cell communication genes expressed by ccRCC cells within the TME. Most of them have been separately mentioned as ccRCC ligands or receptors at the protein level by immunohistochemistry (IHC) in ccRCC tissues, *in vitro* on RCC cell lines^23^^,^^36^^,^^37^^,^^38^^,^^39^^,^^40^^,^^41^ or reported in independent scRNAseq studies.^14^^,^^42^ In addition to recapitulating current knowledge of ccRCC cell ligands and receptors, we discovered 6 novel communication genes (*PLXNB2, LSR, CCL28, NLGN1*, *COL27A1, ALKAL2*) expressed by ccRCC cells. We suggest that all these molecules collectively form the tumor cell-specific vocabulary in ccRCC. Some are involved in immunosuppression (CD70, HHLA2),^30^^,^^31^ angiogenesis (CCL28, VEGFA, SSP1, NLGN1),^24^^,^^43^^,^^44^ cell proliferation and invasion (PLXNB2, LSR, ALKAL2).^45^^,^^46^^,^^47^ Many of these molecules can exert multiple functions, adding to the complexity of the biological interpretation. For example, CD70 can stimulate T cell activation^48^ and induce T cell apoptosis.^31^ Similarly, osteopontin (*SSP1*) has been reported to induce angiogenesis,^43^ stimulate or inhibit cancer cell proliferation depending on the context,^49^ and induce epithelial-mesenchymal transition (EMT).^50^ It therefore remains challenging to assign them a specific biological function, given their pleiotropic nature and their possible context-dependent effects.

This work focused on communication channels specific to ccRCC cancer cells by comprehensively analyzing ligand and receptor expressions from all cell types within the ccRCC TME. Historically, most of the cancer studies were devoted to the analysis of a single crosstalk between two cell types, which has led to the identification of important therapeutic targets such as PD-1 in cancer cell - T cell crosstalk.^51^ The development of next-generation sequencing and single-cell omics technologies increased the amount of communication molecules that could be studied and exploited for therapy or treatment. Some studies inferred cancer cell communication at a large-scale based on bulk tumor RNAseq to identify autocrine cancer cell interactions in leukemia and lymphoma.^52^ Others analyzed the transcriptome of micro-dissected cancer-associated fibroblasts (CAF) and ovarian cancer cells to highlight TGFb/activin A interaction.^53^ Finally, the study of the modulation of CAF transcriptome compared to healthy fibroblasts from scRNAseq data suggested CAF ligands that could promote EMT in head and neck cancer.^12^ Even at the whole transcriptomic scale, most of the studies were limited to analyzing a single cellular crosstalk. Recently, a study analyzed comprehensively cell-cell communication by multiple cell types in Parkinson’s disease and highlighted endothelial cell genes involved in the modulation of cell communication networks.^54^ Here, we applied a comprehensive analysis of cell-cell communication by multiple cell types in the ccRCC TME. By intersecting cell-cell communication analysis and differential expression analysis of cell communication genes, we identified 50 putative interactions specifically used by the cancer cell to interact with other cell types in the TME. The interactions highlighted may lead to the identification of new therapeutic targets.

Despite the power of large-scale systemic analyses, scRNAseq has several limitations. scRNAseq datasets are often generated from a limited number of patients which may not be representative of the disease heterogeneity. In addition, scRNAseq captures fewer genes than bulk RNAseq, mainly due to dropout, which mostly affects low-expressed genes such as cytokines. Hence, important functional genes may be missed by this technique. To overcome these limitations, we applied our cell communication analysis pipeline to three independent scRNAseq datasets, considered only interactions found in at least two datasets to increase the robustness of the results, and retained interactions that were also upregulated in TCGA-KIRC tumors compared to controls. We further checked that already described tumor-specific interactions were also found in the results. Among them, five interactions are currently targeted by approved therapies (*VEGF* and its receptors),^55^ have been already tested in ccRCC (interactions involving *EGFR*, *EDN1/EDNRA*)^25^^,^^56^ or are currently investigated in clinical trials (such as *CD70/CD27* in NCT05420519 and NCT04696731).^57^ This increases confidence in our computational results aimed at identifying novel therapeutic targets, although further experimental validation remains essential. Among the identified interactions, we suggest *EDN1/EDNRB* as a putative novel tumor/endothelial cell interaction, and *ANG/PLXNB2* and *ANG/EGFR* as putative new autocrine loops in ccRCC cancer cells. We propose that these interactions be given greater consideration notably as promising new therapeutic targets which, to our knowledge, have not yet been studied in ccRCC.

In this work, we also showed that angiogenin and its two recently identified receptors, EGFR and PLXNB2,^45^^,^^58^ were upregulated at the protein level by ccRCC cancer cells as compared to their cell type of origin *ex vivo*. Angiogenin upregulation is not restricted to ccRCC and has been described in other cancer types, such as breast, pancreatic, colorectal, and prostate cancers.^59^ EGFR expression has been extensively assessed among solid tumors,^60^ unlike PLXNB2 expression. High PLXNB2 expression has been reported in glioma and breast cancer cell lines,^61^^,^^62^ and has been recently examined in several primary tumor types by IHC.^45^ Angiogenin axis has been poorly studied in the context of ccRCC. Here, we show that angiogenin can regulate ccRCC cell line proliferation, as previously described for endothelial cells,^33^ and breast and prostate cancer cell lines.^45^ Our results revealed that angiogenin down-regulated the secretion of IL-6, IL-8 and MCP-1 pro-inflammatory molecules by cancer cells, suggesting angiogenin could inhibit the inflammation. This result is consistent with another study focusing on an angiogenin-dependent modulation of activated fibroblast phenotype *in vitro,*^35^ where ANG was described as suppressing the inflammatory response through the inhibition of TBK1-mediated NF-κB nuclear translocation. Together, our findings suggest that angiogenin could represent a new therapeutic target in ccRCC. ANG inhibitors (neutralizing antibodies or small chemical compounds blocking angiogenin nucleus translocation) have already been described to inhibit tumor growth *in vitro* and *in vivo,*^45^^,^^59^^,^^63^ but further studies in the context of ccRCC are needed to evaluate their therapeutic potential.

Our results give a new impulse to our understanding of the molecular interactions at play in the ccRCC TME. Importantly, our work demonstrates that large-scale analysis of cell-cell communication can provide new biological insights and help for the identification of new therapeutic targets. This approach could be extended to other cancer types. It could also help monitor the modulation of cell communication networks upon treatment, for example to identify mechanisms leading to immune escape or treatment resistance.

### Limitations of the study

This study uses scRNAseq data to reconstruct communication networks in the TME. However, the spatial organization of the cells is missing. Experimental validations of identified interactions will be important to confirm cellular proximity.

Also, the low representation of some cell types in the scRNAseq data, such as fibroblasts, can be a limitation. CAF have been reported to be very heterogeneous and key modulators of the tumor microenvironment.^64^The use of stromal cell-specific markers in the sorting strategy before sequencing could allow to enrich the sample for stromal populations and deeper characterize cell-cell communication networks, taking into account cell type functional heterogeneity.

Finally, we showed that ANG promotes cancer cell proliferation and suggested that it was mediated by ANG-PLXNB2 and ANG-EGFR interactions. However, we could not investigate the respective contribution of each receptor on cell function. Binding to PLXNB2 and EGFR have both been reported to trigger ERBB2 phosphorylation and to activate similar signaling cascades, such as PI3K/Akt pathway.^45^^,^^62^ Receptor promiscuity and multiple ligands binding the same receptor create major challenges that need to be addressed in future studies, based on novel cellular models.

## STAR★Methods

### Key resources table

**Table:** 

REAGENT or RESOURCE	SOURCE	IDENTIFIER
**Antibodies**		

APC Mouse anti-human CD45 (Clone HI30)	BD	Cat# 561864; RRID: AB_11153499
AF700 Mouse anti-human CD45 (Clone HI30)	BD	Cat# 560566; RRID: AB_1645452
APC-Cyanine7 Mouse anti-human CD45 (Clone 2D1)	BD	Cat# 557833; RRID: AB_396891
BV785 Mouse anti-human CD3 (Clone OKT3)	Biolegend	Cat# 317329; RRID: AB_11219196
Alexa Fluor 700 Mouse anti-human CD3 (Clone UCHT1)	Biolegend	Cat# 300424; RRID: AB_493741
PeCF594 Mouse anti-human CD11c (Clone B-ly6)	BD	Cat# 562393; RRID: AB_11153662
FITC Mouse anti-human CD56 (Clone HCD56)	Biolegend	Cat# 318304; RRID: AB_604100
PerCP/Cyanine5.5 Mouse anti-human CD19 (Clone HIB19)	Biolegend	Cat# 302230; RRID: AB_2073119
Alexa Fluor 700 Mouse anti-human CD14 (Clone M5E2)	BD	Cat# 561029; RRID: AB_396944
BV421 Mouse anti-human CD4 (Clone RPA-T4)	BD	Cat# 562425; RRID: AB_11154417
APC Mouse anti-human CA9 (Clone 303123)	R&D Systems	Cat# FAB2188A; RRID: AB_883864
FITC Mouse anti-human CD31 (Clone WM59)	BD	Cat# 555445; RRID: AB_395838
BUV737 Mouse anti-human CD70 (Clone Ki-24)	BD	Cat# 612856; RRID: AB_2739193
BUV737 Mouse IgG3 isotype control (Clone J606)	BD	Cat# 612854; RRID: AB_2869662
BV785 Mouse anti-human CD13 (Clone WM13)	Biolegend	Cat# 301725; RRID: AB_2734250
PE/Cyanine7 Mouse anti-human EGFR (Clone AY13)	Biolegend	Cat# 352910; RRID: AB_2562159
PE/Cyanine7 Mouse IgG1 isotype control (Clone MOP-C1)	Biolegend	Cat# 400126; RRID: AB_326448
PE anti-human Plexin-B2 (Clone REA626)	Miltenyi	Cat# 130-126-532; RRID: AB_2889732
REA Control - PE antibody	Miltenyi	Cat# 130-104-612; RRID: AB_2661690
Goat anti-human ANG	R&D Systems	Cat# AF265-SP; RRID: AB_2227144
Normal Goat IgG control antibody	R&D Systems	Cat# AB-108-C; RRID: AB_354267
Rabbit anti-human PDZK1IP1	Sigma Aldrich	Cat# HPA014907; RRID: AB_1855166
Rabbit anti-human CA9	Novus	Cat# NB100-417SS; RRID: AB_788423
Alexa Fluor 594 Donkey anti-Goat IgG	Invitrogen	Cat# A32758; RRID: AB_2762828
Alexa Fluor 488 Donkey anti-Rabbit IgG	Invitrogen	Cat# A21206; RRID: AB_2535792
Alexa Fluor 647 Donkey anti-Rabbit IgG	Invitrogen	Cat# A31573; RRID: AB_2536183

**Biological samples**		

Human tumor and juxtatumor samples	Saint-Louis and Bichat Hospitals (APHP)	N/A

**Chemicals, peptides, and recombinant proteins**		

Collagenase I	Sigma Aldrich	Cat# C0130
Hyaluronidase	Sigma Aldrich	Cat# H3506
Liberase	Roche	Cat# 05401020001
DNAse I	Roche	Cat# 1010415900
EDTA	Invitrogen	Cat# 15575-038
Human Serum	Sigma	Cat# H4522-100mL
Fetal Calf Serum	HyClone	Cat# CH30160.03
MEM Non-Essential Amino Acids	Gibco	Cat# 11140050
Pyruvate	Gibco	Cat# 11360070
10% Formalin solution	Sigma Aldrich	Cat# HT501128
Antigen retrieval solution pH9	Invitrogen	Cat# 00-4956-58
Triton	Sigma Aldrich	Cat# X100
Bovine Serum Albumin	Sigma Aldrich	Cat# A7906
Fish gelatin	Sigma Aldrich	Cat# G7041
Glycine	GE Healthcare	Cat# 17-1323-01
DAPI	Invitrogen	Cat# D1306
Prolong Gold Antifade Reagent	Invitrogen	Cat# P36930
DharmaFECT 1 transfection reagent	Dharmacon	Cat# T-2001-01

**Critical commercial assays**		

Zombie Aqua Fixable Viability Kit	Biolegend	Cat# 423101
EasySep RBC Depletion Reagent kit	Stem Cell	Cat# 18170
CD45 microbeads kit	Miltenyi	Cat# 130-045-801
LS columns	Miltenyi	Cat# 130-042-401
Chromium 10X Next GEM Single-cell 3′ Kit (v3.1)	10X Genomics	Cat# 100012
Chromium Next GEM Chip G Single Cell Kit	10X Genomics	Cat# 1000127
Human Angiogenin Flex Set	BD	Cat# 558328
Human VEGFα Flex Set	BD	Cat# 558336
Human IL-6 Flex Set	BD	Cat# 558276
Human IL-8 Flex Set	BD	Cat# 558277
Human MCP-1 Flex Set	BD	Cat# 558287
Human IL-1β Flex Set	BD	Cat# 558279
Human TNFα Flex Set	BD	Cat# 558273
Luminex assay (TGFa, OPN)	R&D System	Cat# LXSAHM

**Deposited data**		

Human reference genome GRCh38-2020-A	Genome Reference Consortium	https://www.ncbi.nlm.nih.gov/grc/human
scRNAseq data	This paper	GSE222703
Public scRNAseq data	Zhang et al.^14^	GSE159115
Public scRNAseq data	Young et al.^23^	Supplementary material of the publication
TCGA-KIRC bulk RNAseq data	The Cancer Genome Atlas Consortium	https://portal.gdc.cancer.gov/

**Experimental models: Cell lines**		

Human: Caki1	HEGP-APHP (Eric Tartour)	N/A
Human: Caki2	Merck	Cat# 93120819-1VL
Human: A498	HEGP-APHP (Eric Tartour)	N/A
Human: 786-O	HEGP-APHP (Eric Tartour)	N/A
Human: RPTEC/TERT1	Saint-Louis Hospital (Ali Amara)	N/A

**Oligonucleotides**		

ON-TARGETplus Human ANG (283) siRNA SMARTpool	Dharmacon	Cat# L-011206-00-0005
ON-TARGETplus Non-targeting Control Pool	Dharmacon	Cat# D-001810-10-05

**Software and algorithms**		

Flowjo version 10	BD	https://www.flowjo.com
R version 4.1.0	https://www.r-project.org/	https://www.r-project.org
Seurat version 4.1.1	Butler et al.^65^	https://satijalab.org/seurat/
10X Cellranger analysis pipeline version 4.0.0	10X Genomics	https://support.10xgenomics.com/single-cell-gene-expression/software/pipelines/latest/using/tutorial_in
DropletUtils version 1.12.3	Bioconductor	https://bioconductor.org/packages/release/bioc/html/DropletUtils.html
scDblFinder version 1.6.0	Bioconductor	https://bioconductor.org/packages/release/bioc/html/scDblFinder.html
scds version 1.8.0	Bioconductor	https://www.bioconductor.org/packages/release/bioc/html/scds.html
Harmony version 0.1.0	Comprehensive R Archive Network	https://github.com/immunogenomics/harmony
Metascape version 2.5.20230501	Zhou et al.^66^	https://metascape.org/
ICELLNET version 1.2.0	Noël et al.^11^	https://github.com/soumelis-lab/ICELLNET/
CellChat version 1.6.1	Jin et al.^32^	https://github.com/sqjin/CellChat
DESeq2 version 1.32.0	Bioconductor	https://bioconductor.org/packages/release/bioc/html/DESeq2.html
ZEN software version 2.3.69.1018	Zeiss	https://www.zeiss.com/microscopy/en/products/software/zeiss-zen.html
Qupath version 0.3.2	Bankhead et al.^67^	https://qupath.github.io
Incucyte base analysis software	Sartorius	https://www.sartorius.com/en/products/live-cell-imaging-analysis/live-cell-analysis-software/incucyte-base-software
FCAP Array version 3.0	BD	Cat# 652099
xPONENT version 4.2.1324.0	Luminex	https://www.luminexcorp.com/xponent/#overview
Prism version 9	GraphPad	https://www.graphpad.com

**Other**		

Source code	This paper	https://github.com/lmassenet-regad/CellComm_ccRCC https://doi.org/10.5281/zenodo.8366431

### Resource availability

#### Lead contact

Further information and requests for resources and reagents should be directed to and will be fulfilled by the lead contact, Vassili Soumelis (vassili.soumelis@aphp.fr).

#### Materials availability

This study did not generate new unique reagents.

#### Data and code availability

• The data generated in this study data have been deposited at Gene Expression Omnibus under the accession number GSE222703. Zhang et al. scRNAseq data are available at GSE159115. Young et al. scRNAseq data are available in the supplementary material of the original publication.^23^ TCGA data are available in the GDC Data Portal website (https://portal.gdc.cancer.gov/). Microscopy data reported in this paper will be shared by the lead contact upon request.
• The source codes for processing all the datasets and generate the figures are available as a GitHub repository (https://github.com/lmassenet-regad/CellComm_ccRCC) and have been deposited on Zenodo (https://doi.org/10.5281/zenodo.8366431).
• Any additional information required to reanalyze the data reported in this paper is available from the lead contact upon request.

### Experimental model and study participant details

#### Human samples

All tissue samples were from patients that underwent partial or total nephrectomy for localized ccRCC at Saint-Louis and Bichat Hospitals. All tumor samples were reviewed by a pathologist to confirm the ccRCC diagnosis, based on tissue histology and CA9 marker positivity. The study design was approved by Saint-Louis Hospital IRB (Institutional Review Board) and all samples were obtained in accordance with the principles of Good Clinical Practice and the Declaration of Helsinki. Each patient signed an informed consent before inclusion to the study. Clinical information of patients used for scRNAseq experiments is provided in Table S7.

#### Cell lines

Caki2 cell line was purchased from Merck (93120819-1VL) and cultured in RPMI 1640 Medium Glutamax (Life Technologies) supplemented with 10% FCS (Hyclone), and 100 U/ml Penicillin/Streptomycin (Gibco). RPTEC/TERT1 cells was kindly given by Ali Amara (Hôpital Saint Louis, Paris) and cultured in DMEM/F12 medium (Gibco, 11320-033) supplemented with hTERT Immortalized RPTEC Growth Kit (ATCC, ACS-4007), and 50 mg/mL Geneticin (Gibco, 10131-035). 786-O, Caki1 and A498 cell lines were kindly given Eric Tartour (Hôpital Européen Georges Pompidou, Paris) and cultured in DMEM/F12 medium (Gibco) for 786-O or DMEM (Gibco) for A498 and Caki1, supplemented with 10% FCS (Hyclone), 100 U/ml Penicillin/Streptomycin (Gibco). All cell lines were cultured at 37°C under humidified 5% CO2 and were regularly negatively tested for mycoplasma contaminations.

### Method details

#### Tissue sample preparation

Tissue samples were mechanically and enzymatically digested in CO2-independent medium (Gibco 18045070) containing 20% Fetal Calf Serum (HyClone). Enzymatic digestion consisted in two rounds of 15 min incubation with agitation at 37°C with 2 mg/mL collagenase I (Sigma #C0130), 2 mg/mL hyaluronidase (Sigma #H3506), 0.1 mg/mL Liberase (Roche #05401020001), and 25 μg/mL DNAse I (Roche #1010415900). Cell suspension was filtered on 100 μm filter and washed with PBS supplemented with 2 mM EDTA (Gibco) and 1% human serum (Sigma). Red blood cells were removed by magnetic isolation using EasySep RBC Depletion Reagent kit (Stemcell #18170) according to the manufacturer’s instructions. For flow cytometry experiments, the cell suspension was stained directly. For scRNAseq samples, the cell suspension was divided into two parts. One part was further enriched in CD45^−^cells by removing CD45^+^ cells using human CD45 microbeads kit (Miltenyi #130-045-801), according to the manufacturer’s instructions. Both cell fractions were then washed and stained directly.

#### Sample staining, flow cytometry and cell sorting

After dissociation, cell suspensions were stained for viability with Zombie Aqua Fixable Viability Kit (Biolegend, #423101) in PBS for 15 min at room temperature. After washing, cells were incubated with surface staining antibodies for 15 min at 4°C in PBS supplemented with 2 mM EDTA and 1% human serum. For flow cytometry experiments, cells were stained with the following antibodies: BV785 anti-CD13 (Biolegend, 301725, 1:50), FITC anti-CD31 (BD, 555445, 1:20), APC-Cy7 anti-CD45 (BD, 557833, 1:40), APC anti-CA9 (R&D Systems, FAB2188A, 1:50), PeCy7 anti-EGFR (Biolegend, 352910, 1:40) or PeCy7 anti-IgG1 (Biolegend, 400426), PE anti-PLXNB2 (Miltenyi, 130-126-532, 1:40) or PE-REA (Miltenyi, 130-104-612), BUV737 anti-CD70 (BD, 612856, 1:40) or BUV737 anti-IgG3 (BD, 612854), BV421 anti-PDL1 (BD, 563738, 1:50) or BV421 anti-IgG1(BD, 562438). Cells were fixed with 1% PFA for 15 min and stored in PBS supplemented with 2 mM EDTA and 1% human serum until analysis. Flow cytometry phenotyping experiments were performed on BD LSR Fortessa Analyzer, and analyzed with FlowJo (BD Biosciences, v10).

For scRNAseq experiments, the first part of cell suspension was stained with the following antibodies: APC anti-CD45 (BD, 561864, 1:20), BV785 anti-CD3 (Biolegend, 317329, 1:100), PE-CF594 anti-CD11c (BD, 562393, 1:100), FITC anti-CD56 (Biolegend, 318304, 1:50), PerCP-Cy5.5 anti-CD19 (Biolegend, 302230, 1:100), AF700 anti-CD14 (BD, 561029, 1:30), BV421 anti-CD4 (BD, 562425, 1:60). The part enriched in CD45^−^cells was stained with APC anti-CA9 (R&D Systems, FAB2188A, 1:50), AF700 anti-CD45 (BD, 560566, 1:20), FITC anti-CD31 (BD, 555445, 1:20), and AF700 anti-CD3 (Biolegend, 300424, 1:40). Cells were sorted on a BD FACS Aria III using FACSDiva v8.0.1. Cell sorting gating strategy is provided in Figures S1A and S1B. Briefly, cells debris and doublets were excluded from FSC/SSC gating. Dead cells were excluded based on viability marker gating. Following viable cell populations were isolated and sorted: 1) From the enriched CD45^−^cell suspension fraction, we sorted CD45^−^CD3^−^cells; 2) From the other cell suspension fraction, we sorted CD45^+^CD3+cells, CD45^+^CD3^−^CD56^+^ cells, CD45^+^CD3^−^CD45^−^CD14^−^CD19^+^ cells, CD45^+^CD3^−^CD45^−^CD19^−^CD14^+^ cells, CD45^+^CD3^−^CD45^−^CD19^−^CD14^−^CD4^+^ cells, CD45^+^CD3^−^CD45^−^CD19^−^CD14^−^cells, and CD45^+^CD3^−^CD45^−^CD19^−^CD14^−^CD11c-CD4^−^cells. The detailed gating strategy for cell sorting is provided in Figures S1A and S1B. Cells were sorted in tubes containing RPMI 1640 Medium Glutamax (Life Technologies) supplemented with 10% FCS (Hyclone), 100 U/ml Penicillin/Streptomycin (Gibco), 1% MEM Non-Essential Amino Acids (Gibco), and 1% Pyruvate (Gibco).

#### scRNAseq data generation and analysis

Single-cell libraries were generated using the Chromium 10X Next GEM Single-cell 3′ Kit (v3.1) according to the manufacturer instructions (10X Genomics). Quality of the libraries was assessed by High Sensitivity D1000 ScreenTape assay (Agilent). Next-generation sequencing was performed on an Illumina NovaSeq 6000. FASTQ files were aligned on the GRCh38-2020-A human reference genome using CellRanger (v4.0.0). Sequencing saturation was greater than 90% for all samples based on the CellRanger output files.

Data analysis was conducted on R (v4.1.0) with Seurat (v4.1.1).^65^ True cells were distinguished from empty droplets using DropletUtils package (v1.12.3). Data were preprocessed to retain cells expressing at least 200 genes, 1000 UMIs, and less than 20% of mitochondrial genes. Cells identified as doublets by scDblFinder (v1.6.0) or scds (v1.8.0) packages were removed. Data were normalized for library size and log2-transformed. Dimensionality was reduced by principal component analysis (PCA) to 2,000 most variable genes, using the variance-stabilizing transformation method. The six samples were integrated using Harmony (v0.1.0) based on the first 50 PC of their respective PCA. Louvain graph-based clustering was conducted to identify cell clusters (resolution of 1). Differential gene expression was analyzed by applying Wilcoxon-rank sum tests with a minimum log2FC of 0.25 and an adjusted p value below 0.05, using Seurat. Cell clusters were annotated based on manual inspection of cluster-specific markers (Table S1, Figure 1C).

Malignant cells cluster from tumoral samples was isolated and reclustered at a resolution of 0.1. Differential gene expression analysis between ccRCC1 and ccRCC2 was conducted as previously described (Table S4), and genes with minimal log2FC of 0.5 and and an adjusted p value below 0.05 were considered for functional enrichment analysis using Metascape^66^ (v2.5.20230501). To identify cancer cell-specific communication genes, pairwise differential expression analyses were conducted between ccRCC2 and each cluster in the tumoral samples except ccRCC1 cluster. The gene selection was based on: 1) a minimal log2FC of 0.25, or 2) with a minimal log2FC of 0.1 and percentage of gene expression less than 10% for the other cluster and a difference between percentage expression greater than 5%. The specific genes were identified as the intersection of the results of differential analyses results of all pairs. Differential gene expression between ccRCC2 and PT from juxtatumoral samples was analyzed by applying Wilcoxon-rank sum tests with a minimum log2FC of 0.25 and an adjusted p value below 0.05 (Table S5).

#### Analysis of scRNAseq public data

Zhang et al.^14^ dataset was retrieved from GEO with the accession number GSE159115 (raw count matrices). Young et al. dataset was retrieved from supplementary materials of the original publication.^23^ For both datasets, only samples from ccRCC patients were considered (Table S7). Data were preprocessed with Seurat using the same filtering parameters than the in-house dataset, normalized for library size and log2 transformed. Dimensionality was reduced by principal component analysis (PCA) as previously described. For Zhang et al. dataset, the 11 samples were integrated in Harmony as previously described and then clustered (Louvain graph-based clustering) at 0.5 resolution. For Young et al. dataset, the 6 samples were integrated using Harmony as previously described and clustered at 0.7 resolution. Cell annotation was performed manually and independently for each dataset. Differential analyses for identification of cluster-specific markers were conducted as previously described. Top cluster-specific markers are provided in respectively Table S8 and Table S9. For Young data, malignant cells were subclustered as previously described and differential gene expression analysis was conducted between ccRCC_2 and PT clusters from the juxtatumoral samples.

#### Expression score of communication molecules in scRNAseq data

We computed the average expression profile of each cluster based on normalized counts of scRNAseq data, grouping cells by clusters and their tissue of origin (tumors or juxtatumors). For each cell individually, global expression scores were computed by adding expression levels of all genes belonging to the same family of molecules according to ICELLNET classification in its ligand-receptor database (Table S3): cytokines (253 genes), immune checkpoints (76 genes), chemokines (73 genes), growth factors (94 genes) and cell adhesion molecules (122 genes). For each family of molecules, the average global expression scores of each cluster in each tissue was considered and centered-reduced as z-scores. Log2 fold changes were computed as the ratio of the average global expression scores between the two tissues, in each cluster independently. Differences in scores were compared using Wilcoxon tests with FDR correction.

#### Cell-cell communication analysis

Ligand-receptor interaction database was manually curated from the literature and extended from its original version^11^ including now 1164 interactions (Table S3). Only cells from tumoral samples were considered for cell-cell communication analysis. An average transcriptomic profile was computed for each cluster, and communication genes expressed by less than 10% of the cluster cells were excluded for the corresponding cluster. Communication scores between cell types were computed using ICELLNET (v1.2.0), as previously described^11^:

(Equation 1) $$S_{C1\to C2}=\sum_{\left(i,j\right)=interaction1}^{interaction\;N}l_{i}^{C1}{.r}_{j}^{C2}$$

where $l_{i}^{C1}$ corresponds to the average expression of ligand i emitted by cell type C1, $r_{j}^{C2}$ is the average expression of receptor j expressed by receiving cell type C2, and N corresponds to the length of the ligand/receptor interaction database (in here, N = 1164).

To identify cancer cell-specific interactions, all ligand-receptor interaction scores were computed between all cluster pairs. Then, for each emitting cell type, results were summarized by adding the contribution with each receiving cell types for one interaction (i,j) as follow:

(Equation 2) $$S_{C}\left(i,j\right)=\sum_{k=cell\;type1}^{cell\;type\;K}l_{i}^{C}{.r}_{j}^{k}$$

where $l_{i}^{C}$ corresponds to the average expression of ligand i emitted by the cell type C, $r_{j}^{k}$ is the average expression of receptor *j* by the receiving cell type *k*, and K is the number of cell types (clusters) in the dataset. $S_{C}\left(i,j\right)$ corresponds to the level of communication via the interaction *(i,j)* between the emitting cell C and all receiving cell types. We filtered $S_{ccRCC2}\left(i,j\right)$ scores to retain those between cancer cells and other cell types that were at least 1.5 times higher than all other $S_{C}\left(i,j\right)$. In original data and Young et al. dataset, ccRCC2 cluster is considered as the cancer cells cluster except in Zhang data where all cancer cells were considered. Interactions were further filtered to retain those including communication molecules upregulated in cancer cells compared to PT from juxtatumors, as already described. CellChat (v1.6.1) was run using its defaults parameters when not precised and default human ligand-receptor interactions. Gene expression average was computed as 10% truncated means.

#### TCGA-KIRC analysis

TCGA-KIRC raw bulk transcriptomic profiles were downloaded from GDC Data Portal website (https://portal.gdc.cancer.gov/), selecting 538 primary tumors and 72 normal tissue samples. Counts were normalized using DESeq2 (version 1.32.0), and center-reduced for each gene (scaling). Scaled counts were were used to compute expression product of selected ligand-receptor pairs, for each sample. Comparisons of expression products between tumors and healthy controls were assessed using applying Wilcoxon-rank sum tests and Bonferroni correction.

#### Tissue immunofluorescence and microscopy

Tumors and juxtatumors were fixed 24 h in 10% formalin solution (Sigma, HT501128), stored in PBS until paraffin embedding, and cut into 4 μm sections. Paraffin was removed by 2 times 5 min incubation in xylene (VWR, 28975.291) and rehydrated by successive baths containing 100%, 70%, 50%, 0% ethanol (Merck). Antigen retrieval was performed at pH 9 (Invitrogen, 00-4956-58) for 20 min at 95°C. Sections were permeabilized with 0.5% Triton (Sigma, X100) for 10 min and incubated overnight at 4°C with Goat anti-ANG (R&D Systems, AF265-SP, 5 μg/mL), or its corresponding isotype Goat IgG (R&D Systems, AB-108-C) and Rabbit anti-PDZK1IP1 (Sigma, HPA014907, at 0.3 μg/mL) for juxtatumors or Rabbit anti-CA9 (Novus Bio, NB100-417SS, 1 μg/mL) for tumors, in a blocking solution containing 2.5% Bovine Serum Albumin (Sigma, A7906), 0.1% fish gelatin (Sigma, G7041) and 100 mM glycine (GE Healthcare, 17-1323-01). Tissue sections were washed and incubated 2 h with Alexa Fluor 594 Donkey anti-Goat IgG (Invitrogen, A32758, diluted 1:1500) and Alexa Fluor 488 Donkey anti-Rabbit IgG (Invitrogen, A21206, diluted 1:1500) or Alexa Fluor 647 Donkey anti-Rabbit IgG (Invitrogen, A31573, diluted 1:1500) secondary antibodies at room temperature. Tissue sections were stained in DAPI (Invitrogen, D1306, 2.5 μg/mL) for 5 min at room temperature. Slides were mounted using Prolong Gold Antifade Reagent (Invitrogen, P36930). Images were acquired on a LSM800 confocal microscope (Zeiss) and processed with ZEN software (Zeiss, v2.3.69.1018).

#### Immunofluorescence image quantification

Quantification of the images was performed on Qupath^67^ (0.3.2) to determine cell marker+, ANG+, and cell marker+ANG+ cells. Cell marker was CA9 to identify cancer cells (in tumors), and PDZK1IP1 for proximal tubules (in juxtatumors). Full image annotations were generated. Within the annotations, cells were detected based on DAPI nuclear staining (settings differed between healthy and tumor samples to reflect the differences in the nuclear staining and size: Healthy, threshold: 3500, cell expansion: 6 μm; Tumor, threshold: 3000, cell expansion: 7 μm). Duplicate training images for ANG and cell marker channels were generated for each sample image. On the respective training image, classifiers were trained by manually selecting a representative amount of positive and negative cells. These classifiers were combined into a composite classifier of ANG and cell marker and applied to the respective sample image to determine marker positivity on the DAPI determined cells.

#### Flow cytometry on cell lines

Cell lines were detached with Trypsin 0.05% EDTA (Gibco, 11580626) and stained for viability with Zombie Aqua Fixable Viability Kit (Biolegend, 423101) in PBS for 15 min at room temperature. After washing, cells were incubated with BUV737 anti-CD70 (BD, 555834), PE-Cy7 anti-EGFR (Biolegend, 352909), or PE anti-PLXNB2 (Miltenyi, 130-126-566) surface staining antibodies or their corresponding isotype for 15 min at 4°C in PBS supplemented with 2 mM EDTA and 1% human serum.

#### siRNA and cell proliferation monitoring

For ANG silencing, cells were transfected over 3 days with 50 nM of ON-TARGETplus Human ANG (283) siRNA SMARTpool (Dharmacon, L-011206-00-0005) or 50 nM ON-TARGETplus Non-targeting Control Pool (Dharmacon, D-001810-10-05), using DharmaFECT 1 transfection reagent (Dharmacon, T-2001-01) and according to manufacturer’s instructions. Pooled siRNA containing 4 different target sequences was used to eliminate the possibility of off-target effects. After 3 days, cells were counted and seeded at 5000 cells per well for Caki2, A498 and Caki1 cells or 2500 cells per well for 786-O in 96-well plates, in their respective culture medium containing 0.5% FBS. Proliferation was monitored over 3 days by Incucyte S3 (Sartorius). 5 images of wells were taken every 2 h (10X objective), and cell confluency was computed from the phase images by automatic detection of cells algorithm on the Incucyte base analysis software.

#### Cell culture supernatants analysis

Cell lines supernatants were recovered after 3 days of proliferation and stored at −20°C until analysis. ANG (BD 558328), VEGFα (BD 558336), IL-6 (BD 558276), IL-8 (BD 558277), MCP-1 (BD 558287), IL-1β (BD 558279), and TNFα (BD 558273) levels were assessed by Cytometry Bead Array on a BD LSR Fortessa, whereas TGFα, and OPN concentrations were measured by Luminex assay (R&D Systems, LXSAHM) on a Luminex Magpix instrument, according to manufacturer’s instructions.

### Quantification and statistical analysis

Statistical analyses were performed using GraphPad Prism (v9) and R (v4.1.0). For scRNAseq data analysis, differential gene expression analyses (Tables S1, S2, S4, S5, S6, S8, and S9; Figures 2C–2E) were performed by applying Wilcoxon-rank sum tests and Bonferroni correction. Comparison of communication molecule enrichment scores were performed by applying Wilcoxon-rank sum tests and FDR correction (Figure 1E). Comparison of ligand-receptor expression product in bulk RNAseq data were performed by applying Wilcoxon-rank sum tests and Bonferroni correction (Figure S4D). Kruskal–Wallis tests combined with a Dunn’s post-hoc were used for multiple comparisons of unpaired non-parametric data (Figures 2F and 4F). Mann–Whitney tests were used for two-group comparisons of unpaired non-parametric data (Figures 4D and 4E). Wilcoxon signed rank tests were used for two-group comparisons of paired non-parametric data (Figures 5A and 5C). Repeated measures two-way ANOVA with Geisser Greenhouse correction combined with a Šidák post hoc was used for multiple comparison of cell line proliferation using siRNA (Figure 5B; Figure S6). Data are presented as mean values ±SEM, unless otherwise indicated. When not provided as values, p values are represented by range: ns (non-significant) > 0.05, ∗ <0.05, ∗∗ <0.01, ∗∗∗ <0.001, ∗∗∗∗ <0.0001.

## Supplemental information

Document S1. Figures S1–S6

Table S1. Differential expression analysis of original scRNAseq data at a resolution of 1, related to Figure 1 and STAR Methods

Table S2. Number of cells per cluster per tissue in the original scRNAseq data, related to Figure 1

Table S3. Update of the ICELLNET ligand/receptor database, related to STAR Methods

Table S4. Differential expression analysis between ccRCC1 and ccRCC2 cells in original scRNAseq data, related to Figure 2 and STAR Methods

Table S5. Differential expression analysis of communication molecules between ccRCC2 and proximal tubules from juxtatumors, related to Figure 3 and STAR Methods

Table S6. List of cancer cell-specific interactions found in each dataset and results of in silico validations using CellChat and TCGA-KIRC cohort, related to Figure 3 and STAR Methods Column 1, interaction name; column 2, direction of communication; columns 3–5, datasets were the interaction was found to be cancer cell-specific; column 6, whether the interactions was identified using CellChat; columns 7–8, ligand-receptor expression product in TCGA-KIRC tumors compared to controls.

Table S7. Clinical information of patients included in original and publicly available scRNAseq datasets, related to Figure 1 and STAR Methods

Table S8. Differential expression analysis of Young et al. dataset at a resolution of 0.7, related to STAR Methods

Table S9. Differential expression analysis of Zhang et al. dataset at a resolution of 0.5, related to STAR MethodsMain figure titles and legends
