# Supplementary material for: Large-scale analysis of cell-cell communication reveals angiogenin-dependent tumor progression in clear cell renal cell carcinoma
Source: iScience. 2023 Oct 31;26(12):108367. doi: 10.1016/j.isci.2023.108367 (PMC10663819; doi:10.1016/j.isci.2023.108367)
Supplement: Document S1. Figures S1–S6 [file mmc1.pdf]

iScience, Volume *26*

Supplemental information

Large-scale analysis of cell-cell communication reveals angiogenin-dependent tumor progression in clear cell renal cell carcinoma

Lucile Massenet-Regad, Justine Poirot, Margaret Jackson, Caroline Hoffmann, Elise Amblard, Fanny Onodi, Fatiha Bouhidel, Malika Djouadou, Idir Ouzaid, Evanguelos Xylinas, Jasna Medvedovic, and Vassili Soumelis

SUPPLEMENTAL FIGURES

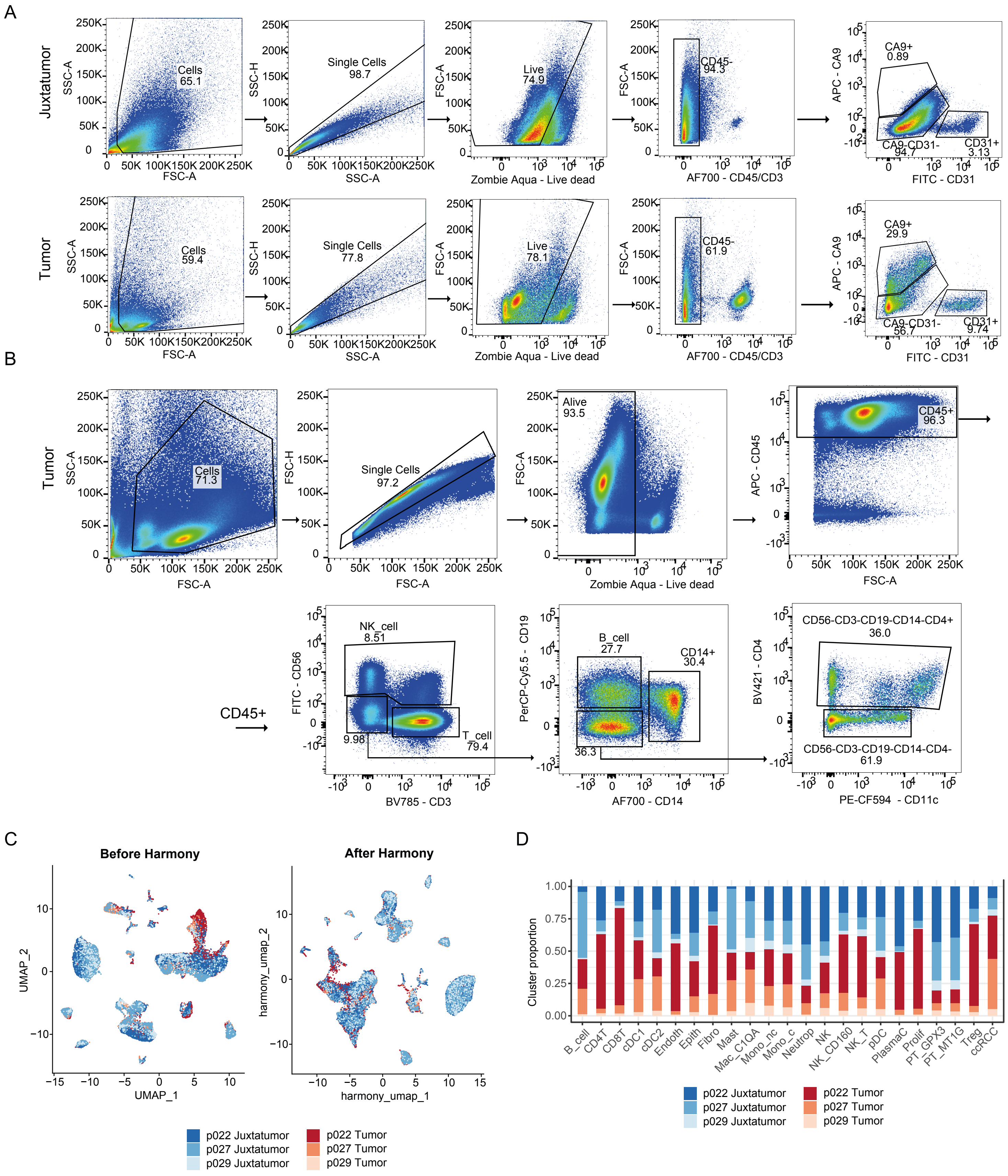

Figure S1: Single-cell RNAseq data generation and analysis of ccRCC tumors and juxtatumors. Related to Figure 1. **A)** Gating strategy for sorting of CD45- cell population starting from CD45- enriched cell suspension, after tumoral (bottom) or juxtatumoral (top) tissue dissociation. CA9 marker is a widely used ccRCC cancer cell marker. **B)** Gating strategy for sorting of CD45+ cell populations starting from a ccRCC tumor-dissociated cell suspension.**C)** Uniform Manifold Approximation and Projection (UMAP) visualization of the scRNAseq data colored by sample of origin (patient and tissue type), before (left) and after (right) batch correction using Harmony. **D)** Barplot visualization of the cell type frequency (vertical axis) of each cluster/cell-type (horizontal axis) in each sample (color).

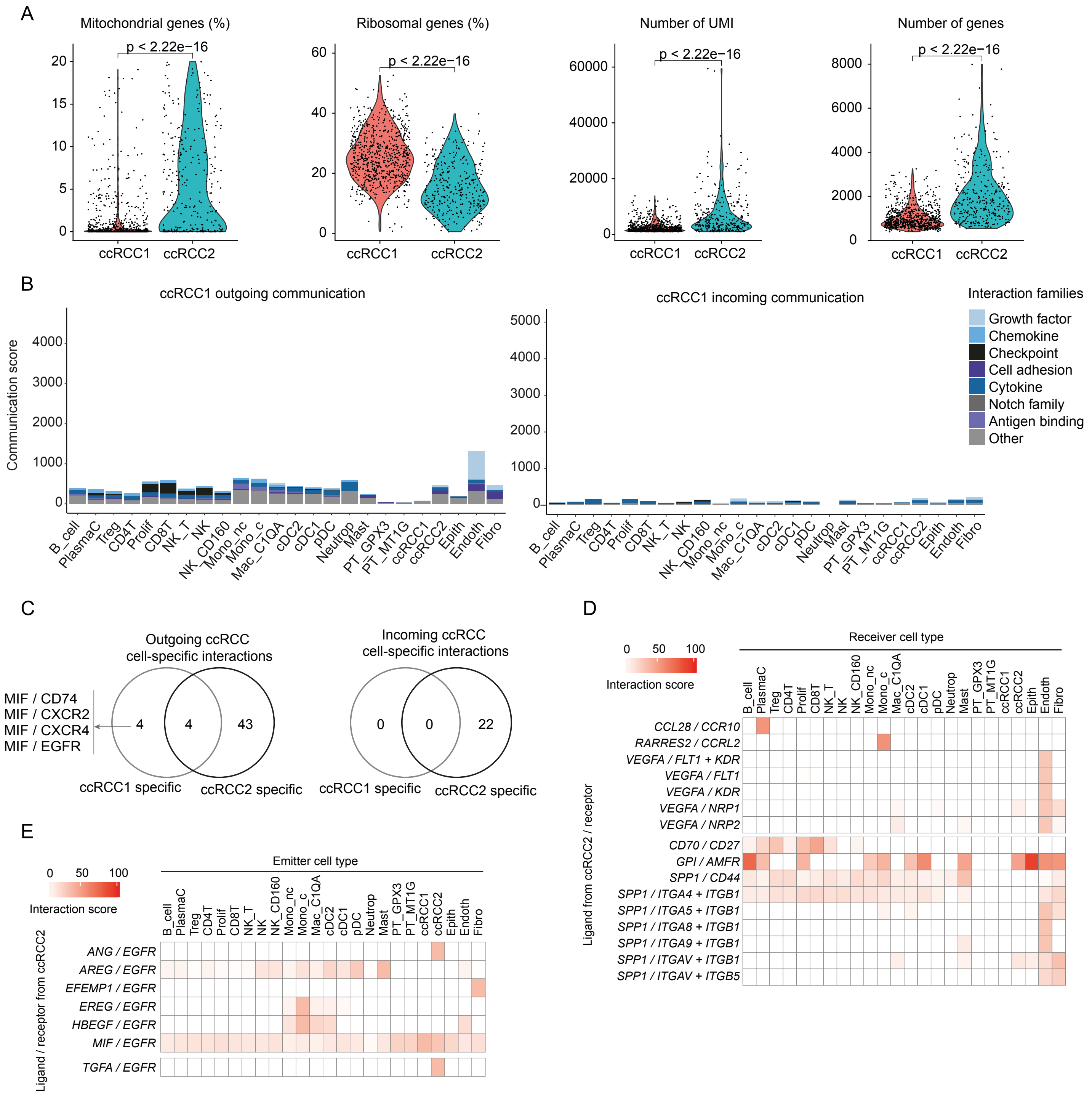

Figure S2: Extended comparison of ccRCC1 and ccRCC2 cancer cells. Related to Figure 2 and Figure 3. **A)** Proportion of mitochondrial genes, ribosomal genes, number of UMI and number of genes for each cancer cell subcluster. The scores were compared by Wilcoxon tests. UMI=unique molecular identifier. **B)** Barplot representing global communication scores between ccRCC1 and the other cell types included in the scRNAseq dataset. (left) represents the outgoing communication scores, meaning ligand expressed by ccRCC1 and receptors expressed by the other cell types. Conversely, (right) represents the incoming communication scores. The contribution of each family of molecules to the communication scores is represented by the color code. **C)** Comparison of cancer cell-specific ligand/receptor interactions identified with ccRCC1 (grey circle) or ccRCC2 (black circle) cancer cells. **D-E)** Outgoing communications scores (D), and incoming communications scores (E) of ccRCC2-specific interactions identified in the original dataset. The heatmap displays the interaction score (from 0 to 100) between ccRCC1 and the other cell type (horizontal axis).

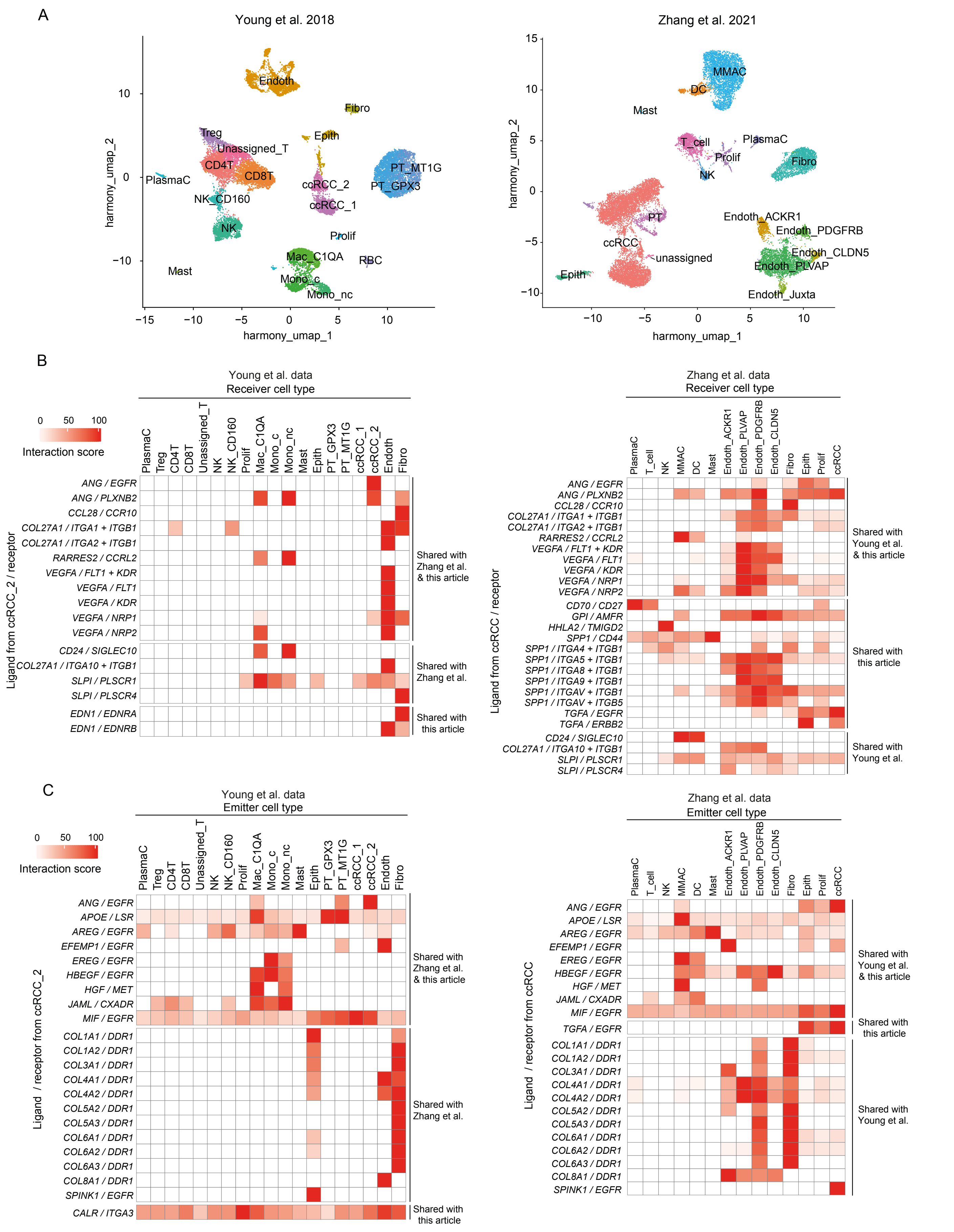

Figure S3: Identification of cancer cell-specific communication channels in public datasets. Related to Figure 3. **A)** Uniform Manifold Approximation and Projection (UMAP) visualization of two public scRNAseq datasets (left: Young et al., right: Zhang et al.), colored according to clusters, and manually annotated. PT: proximal tubule; ccRCC: cancer cell; Mono_c: classical monocyte; Mono_nc: non classical monocyte; Mac_C1QA: macrophage; Epith: epithelial cell; Fibro: fibroblast; Endoth: endothelial cell; NK: natural killer; PlasmaC: plasma cell; RBC: red blood cell, Prolif: proliferating cell, Mast: mast cell; Treg: T regulatory lymphocyte; CD4T: CD4+ T lymphocyte; CD8T: CD8+T lymphocyte; MMAC: monocyte and macrophage; DC: dendritic cell. **B-C)** Heatmaps representing outgoing (B) or incoming (C) cancer cell-specific interactions shared by at least 2 scRNAseq datasets. The heatmaps display the interaction score between the cancer cells and an other cell type (horizontal axis), according to Young et al. data (left) or Zhang et al. data (right).

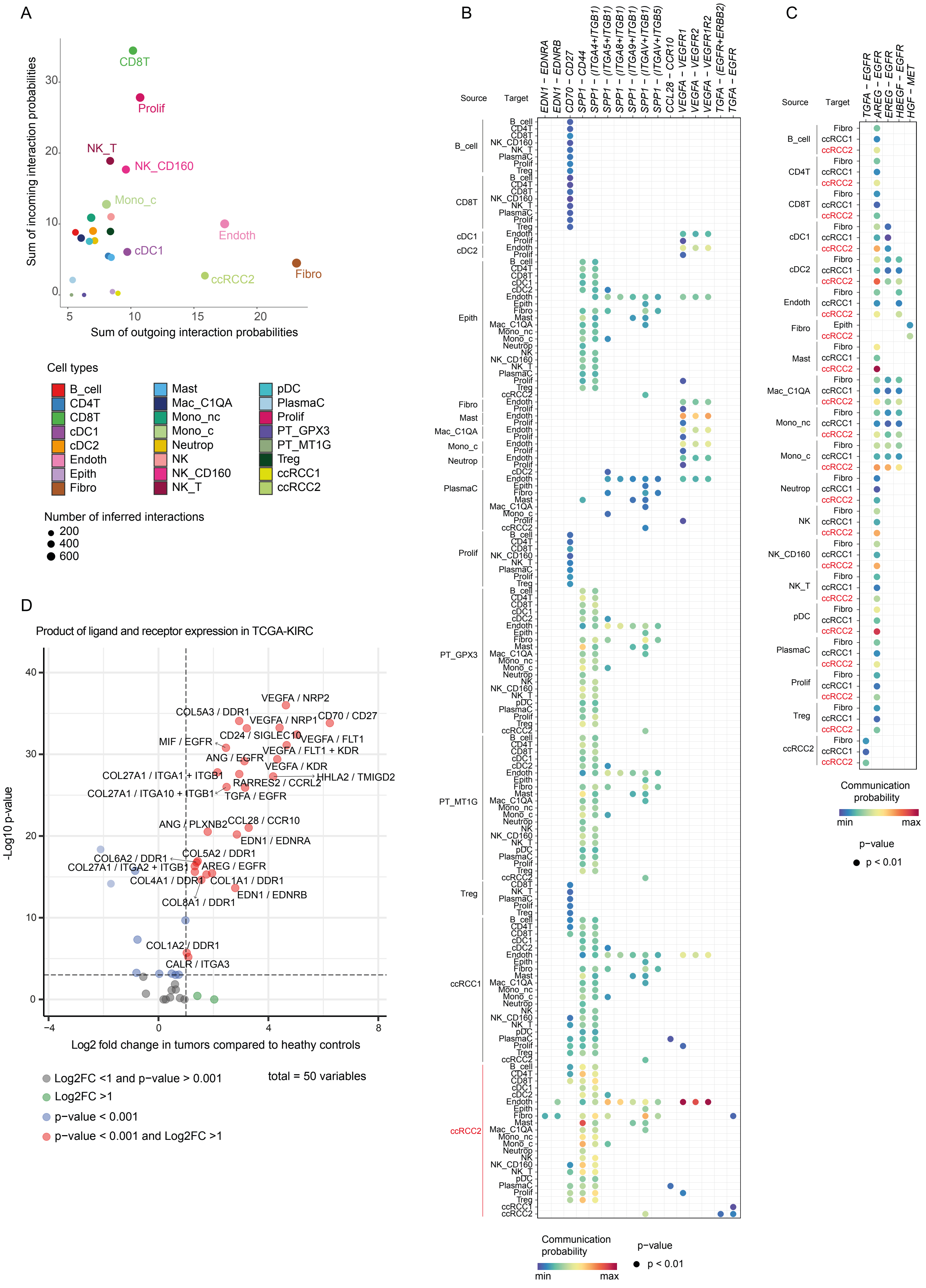

Figure S4: In silico validation of cancer cell-specific interactions. Related to Figure 3. **A)** Scatter plot displaying the

sum of incoming communication probabilities (computed by CellChat) and the sum of outgoing communication probabilities (computed by CellChat), for each cell type. Size of the dot is proportional to the number of inferred ligand-receptor interactions. **B-C)** Outgoing (B) and incoming (C) communication probabilities computed by CellChat of selected ligand-receptor interactions. Color corresponds to value of communication probabilities, and only significant interactions ($p<0.01$) are represented. **D)** Volcano plot representing the product of ligand-receptor pairs expression in TCGA-KIRC tumors relative to their expression in heathy control tissues (log2FC, horizontal axis), and the corresponding adjusted p-value (-log10, vertical axis) obtained by applying Wilcoxon-rank sum tests and Bonferroni correction. Colors indicate ligand-receptor pairs with expression product overexpressed in tumors (greater than one-fold change in expression (log2 value), in red) or with lower fold change expression (in blue), or no significant changes in expression (in grey).

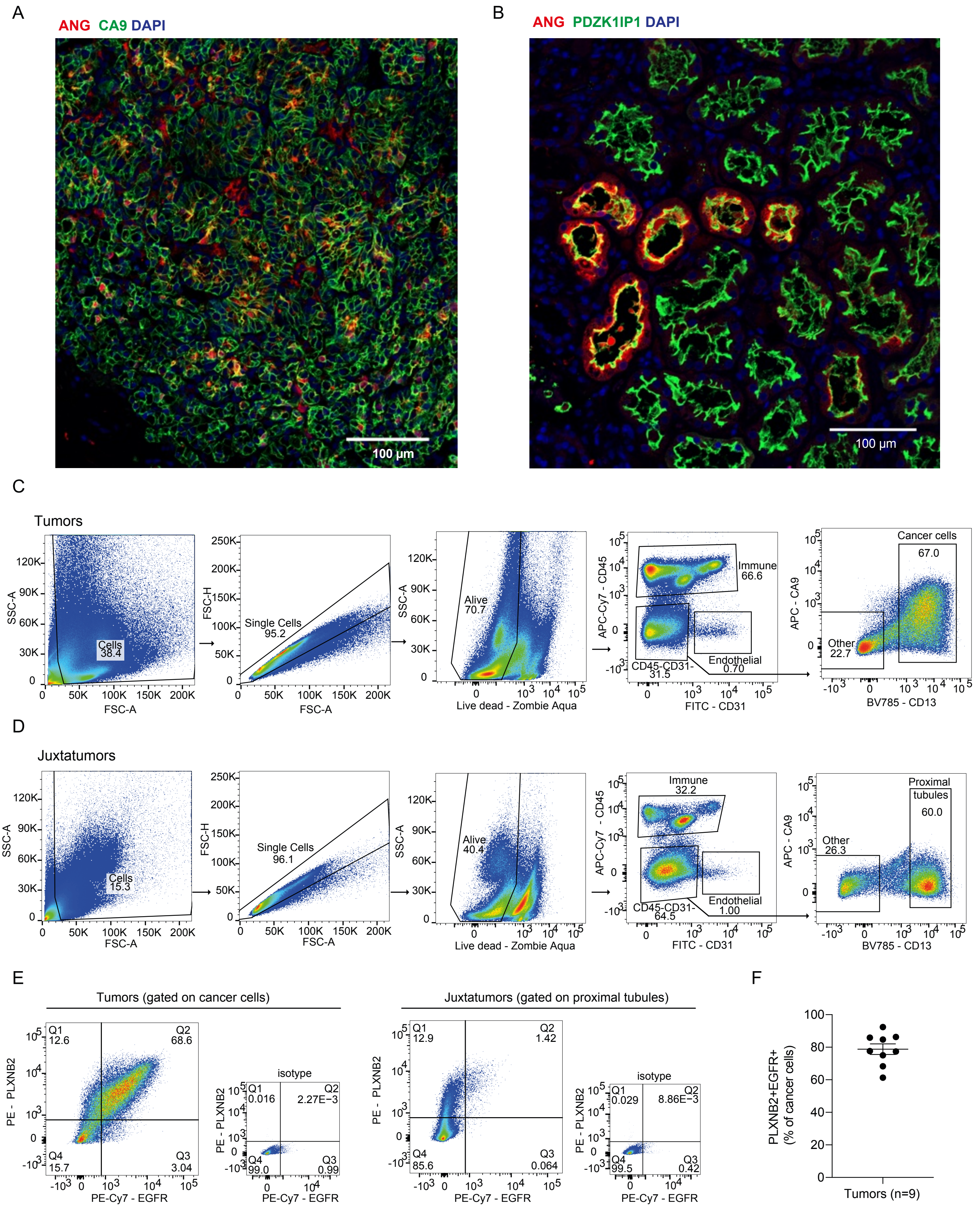

Figure S5: Expression of angiogenin and its receptors by ccRCC cancer cells. Related to Figure 4.
A-B) Representative immunofluorescence images used for image quantifications from ccRCC tumor (A) or juxtatumors (B), showing distribution of ANG (red), CA9 in tumors or PDZK1IP1 in juxtatumors (green), and cell nucleus in DAPI (blue). Scale bar corresponds to 100 µm. **C-D)** Gating strategy of tumor-(C) or juxtatumor-(D) dissociated cell suspension to measure PLXNB2 and EGFR expression in several cellular compartments (Immune: CD45+; Endothelial: CD45-CD31+; Cancer cell/proximal tubule: CD45-CD31-CD13+; Other: CD45-CD31-CD13-). **E)** Representative flow cytometry dot plots showing PLXNB2 and EGFR expression of cancer cells in ccRCC tumors (left) and proximal tubules in juxtatumors (right). Smaller dot plots show unspecific staining with EGFR and PLXNB2 using corresponding isotypes on the same sample. **F)** Proportion of cancer cells co-expressing PLXNB2 and EGFR in ccRCC tumors (n=9 patients). Data are presented as mean values ± SEM.

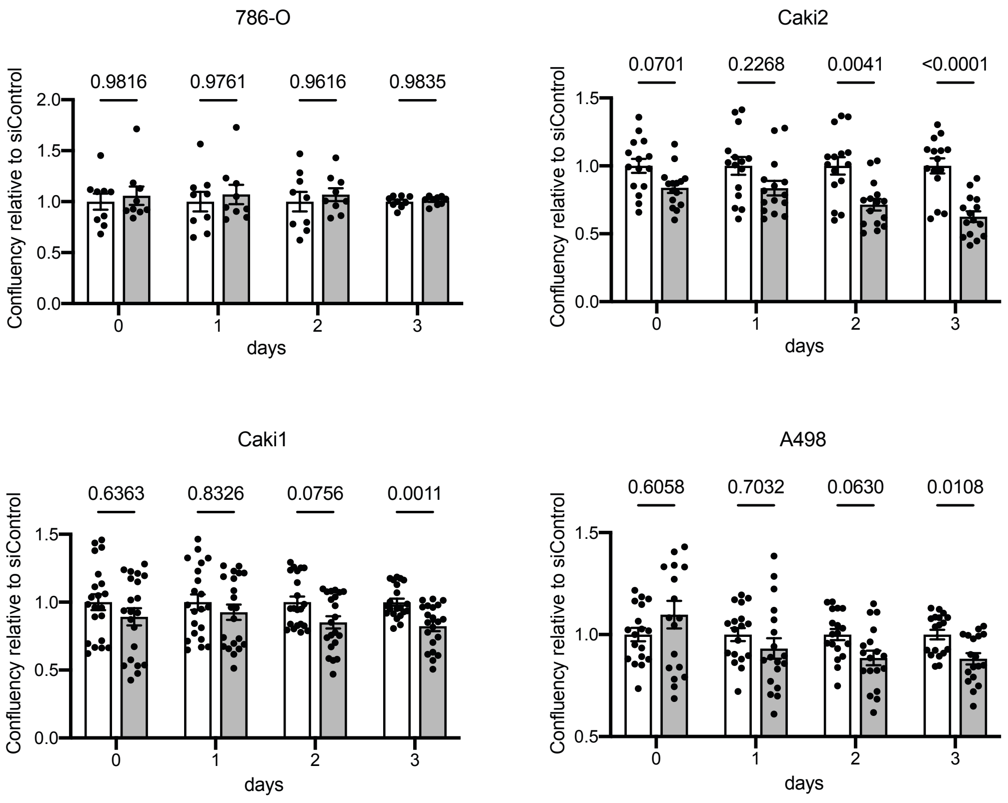

Figure S6: Cancer cell proliferation after transfection with siRNA against ANG (siANG) or control condition (siControl). Related to Figure 5. Compared to Figure 5B, the confluency measurements are normalized by the average confluency measured for siControl condition, for each time point separately. Each independent experiments were run in triplicates (n=6 independent experiments for A498 and Caki2, n=7 for Caki1, and n=3 for 786-O). Data are presented as mean values ± SEM. Conditions were compared using repeated measures two-way ANOVA with Geisser Greenhouse correction combined with a Šidák post hoc.

SUPPLEMENTARY TABLES LEGENDS

Table S1: Differential expression analysis of original scRNAseq data at a resolution of 1. Related to Figure 1 and STAR Methods.

Table S2: Number of cells per cluster per tissue in the original scRNAseq data. Related to Figure 1.

Table S3: Update of the ICELLNET ligand/receptor database. Related to STAR Methods.

Table S4: Differential expression analysis between ccRCC1 and ccRCC2 cells in original scRNAseq data. Related to Figure 2 and STAR Methods.

Table S5: Differential expression analysis of communication molecules between ccRCC2 and proximal tubules from juxtatumors. Related to Figure 3 and STAR Methods.

Table S6: List of cancer cell-specific interactions found in each dataset and results of in silico validations using CellChat and TCGA-KIRC cohort. Column 1, interaction name; column 2, direction of communication; columns 3-5, datasets were the interaction was found to be cancer cell-specific; column 6, whether the interactions was identified using CellChat; columns 7-8, ligand-receptor expression product in TCGA-KIRC tumors compared to controls. Related to Figure 3 and STAR Methods.

Table S7: Clinical information of patients included in original and publicly available scRNAseq datasets. Related to Figure 1 and STAR Methods.

Table S8: Differential expression analysis of Young et al. dataset at a resolution of 0.7. Related to STAR Methods.

Table S9: Differential expression analysis of Zhang et al. dataset at a resolution of 0.5. Related to STAR Methods.
